# Molecular Regulation and Intermolecular Chemistry in Gel Polymer Electrolytes for High‐Voltage Lithium Batteries

**DOI:** 10.1002/advs.202417169

**Published:** 2025-04-09

**Authors:** Qingjie Zhou, Mengxue He, Shuyang Gao, Wangshu Hou, Yulin Ma, Hua Huo, Chunyu Du, Geping Yin, Pengjian Zuo

**Affiliations:** ^1^ College of Chemistry and Molecular Sciences Henan University Kaifeng 475004 China; ^2^ State Key Laboratory of Space Power‐Sources School of Chemistry and Chemical Engineering Harbin Institute of Technology No.92 West‐Da Zhi Street Harbin 150001 China

**Keywords:** gel polymer electrolyte, high‐voltage compatibility, intermolecular interactions, Li⁺ transport kinetics, molecular regulation

## Abstract

Gel polymer electrolyte (GPE) has garnered widespread attention in the field of **lithium** batteries because of its low interfacial impedance, high thermal stability, and flexibility. However, the high‐voltage compatibility and Li^+^ transport kinetics of GPE have yet to meet the requirements of future high‐energy secondary battery systems. In this regard, a comprehensive and insightful review of high‐voltage lithium batteries with GPE has attracted significant attention, focusing on molecular design and intermolecular interactions. Molecular regulation involves customizing the polymer matrix, solvent, additive, and Li salt, while intermolecular interactions encompass hydrogen bond interactions, Lewis acid‐base interactions, electrostatic interactions, and π–π stacking interactions. Besides, strategies to enhance the stability of the cathode electrolyte interphase and Li^+^ transport kinetics are summarized. It is hoped that this review will provide a deeper understanding of the direct regulation of GPE at the molecular level, further accelerating the commercialization of GPE in high‐energy secondary lithium batteries.

## Graphical abstract

In this review, from the perspective of molecular design and intermolecular interactions, polymer matrices, solvents, and Li salts used in high‐voltage gel polymer electrolytes (GPEs) are discussed to explore approaches for improving high‐voltage stability. Additionally, strategies for regulating interphase chemistry and facilitating Li^+^ transport kinetics within GPEs are proposed.

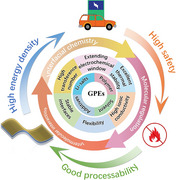

## 1 Introduction

High‐energy and high‐safety lithium batteries have attracted great attention due to the rapidly growing demand for portable electronic devices, electric vehicles, and energy storage systems in recent years.^[^ ^1^ ^]^ To promote the energy density of lithium batteries, the most effective strategies involve elevating the upper cut‐off voltage of the most promising cathodes including lithium‐rich layered oxides, nickel‐rich layered oxides, and lithium cobalt oxides.^[^ ^2^ ^]^ Taking one of the most common battery chemistries in the lithium metal batteries (LMBs), Li||LiNi_x_Co_y_Mn_1‐x‐y_O_2_, (hereinafter, Li||NCM), as an example, boosting the upper cut‐off voltage (> 4.5 V) allows the Ni‐rich NCM cathode to show an additional capacity of ≈15%, as more Li is utilized in each charge/discharge cycle. This contributes to the increase in energy density for LMBs.^[^ ^3^ ^]^ However, the commercial liquid electrolyte (LE) with low oxidative stability (≈4.3 V vs Li^+^/Li) cannot sustain high‐voltage cathodes, triggering the fast deterioration of cycling performance.^[^ ^4^ ^]^ In addition, boosting the upper cutoff voltage inevitably aggravates cycling performance due to other violent side reactions, including cation mixing and reconstruction within the positive electrode materials, oxygen generation, structural cracks, and transition metal dissolution.^[^ ^5^ ^]^ Moreover, thermal runaway under abuse conditions such as overcharging, overheating, and short‐circuiting can also lead to safety hazards like fire and explosion accidents throughout the entire life cycle of the batteries. Consequently, increasing the energy density of lithium batteries and enhancing safety performance have become urgent priorities.^[^ ^6^ ^]^ To address these challenges, solid‐state electrolytes (SSEs) have been proposed and developed.^[^ ^7^ ^]^ Compared with inorganic and polymer electrolytes, gel polymer electrolytes (GPEs), containing polymer matrices, Li‐salts, additives, and solvents, are considered among the best candidates to achieve high‐energy lithium batteries, benefiting from intimate electrode/electrolyte interfacial contact and inherent safety.^[^ ^8^ ^]^

**Figure 1:**
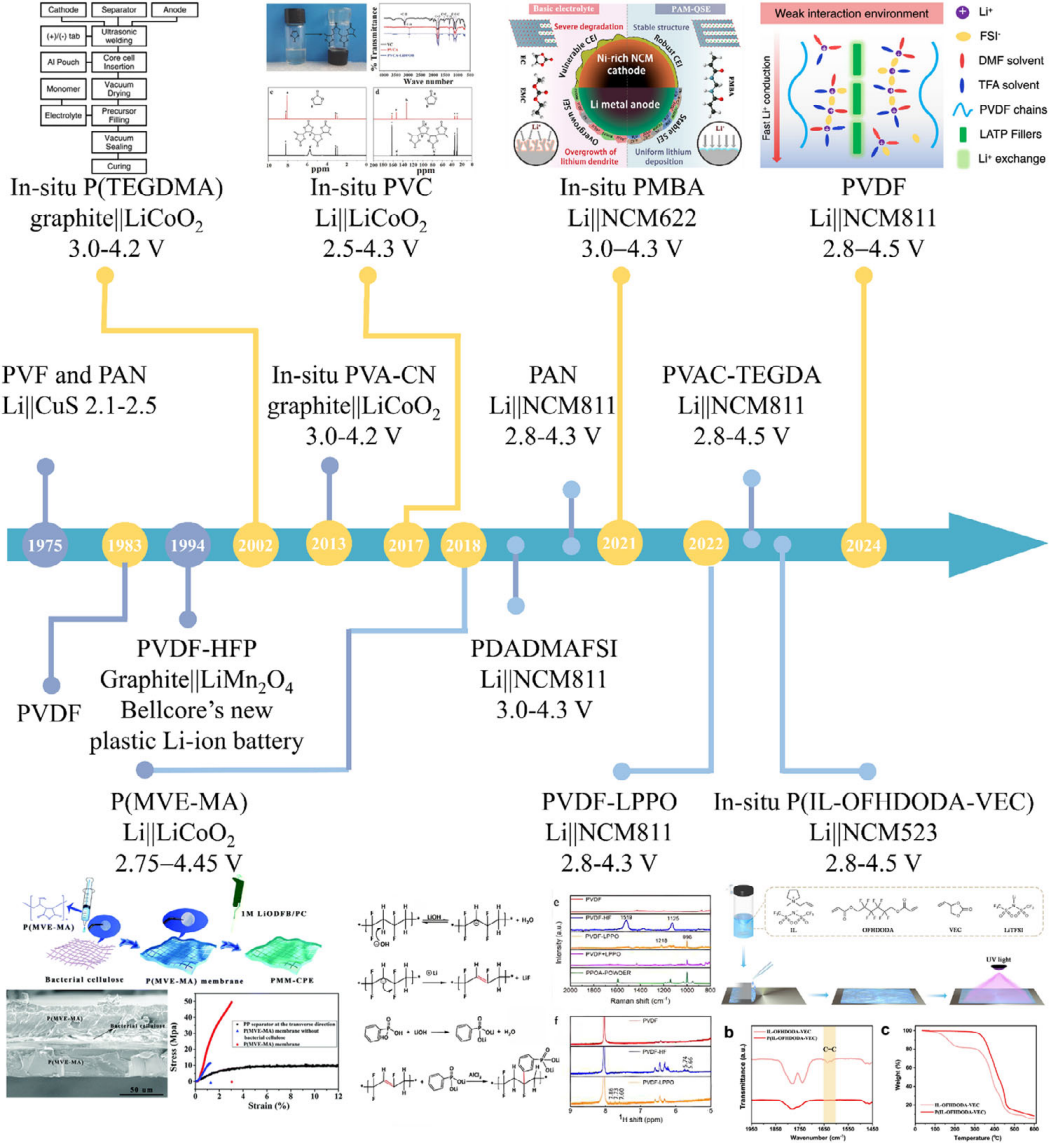
The development timeline of GPE.

**Figure** **1** provides a concise overview of key milestones in the field of GPE.^[^ ^9^ ^]^ In 1975, Perche demonstrated the idea of plasticizing a polymer with an aprotic solution containing an alkali metal salt, in which the organic solution of the alkali metal salt remained trapped within the polymer matrix.^[^ ^9a^ ^]^ Subsequently, poly(vinylidene fluoride) (PVDF)‐based polymers were developed as hosts for GPEs.^[^ ^9b^ ^]^ After two decades, Bellcore technology carried out technical innovation and reported graphite||LiMn_2_O_4_ cells based on a poly(vinylidene fluoride‐co‐hexafluoropropylene) (PVDF‐HFP) copolymer matrix containing a solution of lithium hexafluorophosphate (LiPF_6_) in ethylene carbonate/dimethyl carbonate (EC/DMC).^[^ ^9c^ ^]^ Especially in 2002, in situ polymerized GPE was constructed inside lithium batteries, alleviating the issue of interface compatibility.^[^ ^9d^ ^]^ Subsequently, large amounts of unsaturated monomers for in situ polymerization have been developed with the advancement of high‐voltage lithium batteries. Certain classes of GPEs with various chemistries have achieved high Li⁺ transference numbers and ion conductivities on the order of 1×10^−3^ S cm^−1^ at room temperature, comparable to the ion conductivity of commercial LEs. The oxidative resistance of the electrolytes plays an important role in determining the electrochemical performance of high‐energy lithium batteries, and numerous electrolyte compositions have been developed to meet the demands of aggressive high‐voltage cathodes. Substantial work has clarified that both the components of GPEs and intermolecular interactions determine their high‐voltage stability. In this review, we elucidate how elevated upper cut‐off voltages aggravate cycling performance in lithium batteries from the perspective of electrolyte oxidative decomposition and discuss what can be done with electrolytes to suppress these degradation processes at the molecular regulation and intermolecular chemistry levels.

### 1.1 Frontier Orbital Energy Levels: Theoretical Basis to Tune High‐Voltage Compatibility

**Figure 2:**
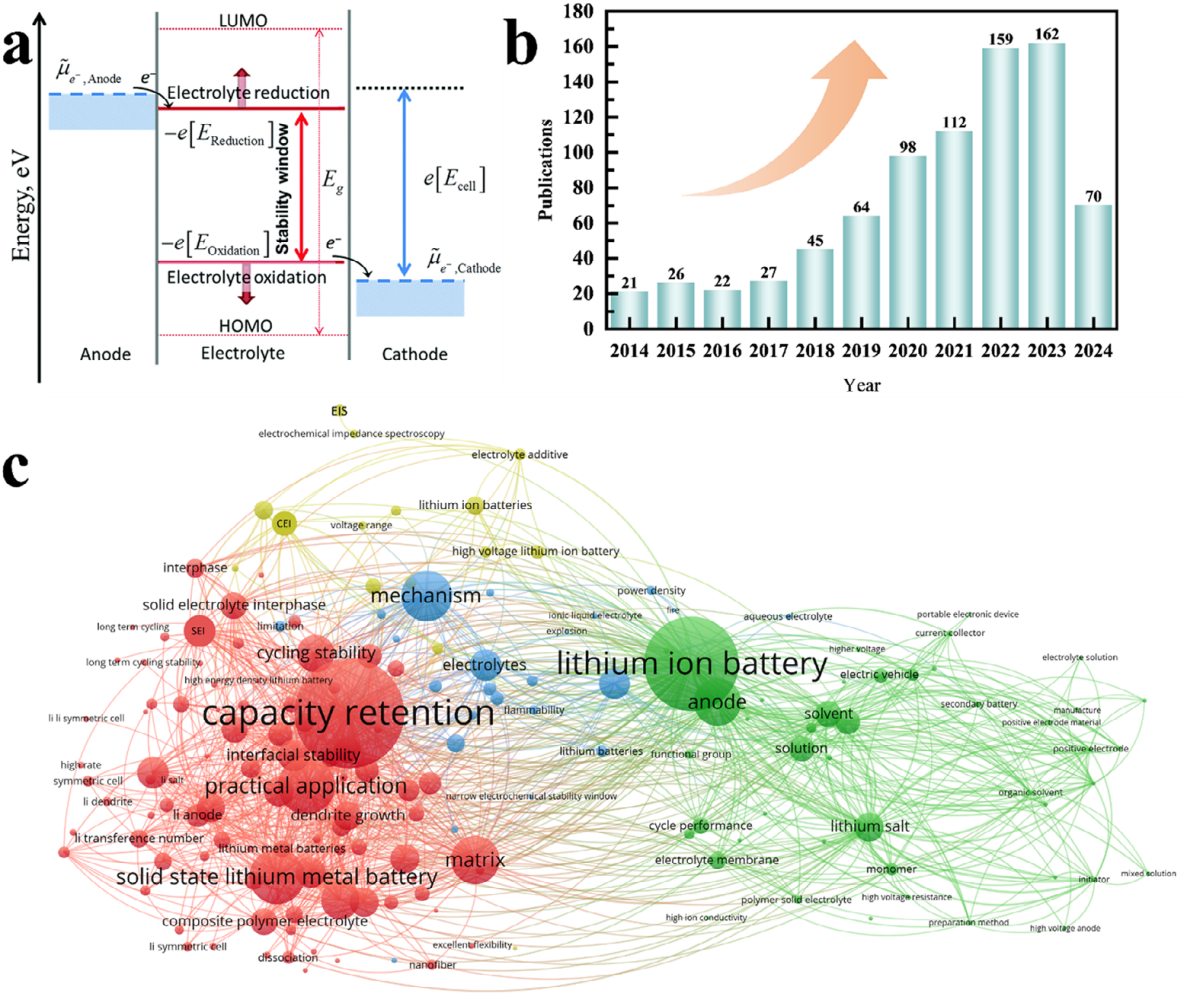
a) The negative and positive potential limits for the electrolyte stability and energy levels of HOMO and LUMO. Reproduced with permission.^[^ ^11^ ^]^ Copyright 2008, RSC Publishing. b) The number of published papers on high‐voltage polymer electrolyte lithium batteries and high‐voltage quasi‐solid‐state lithium batteries, according to Web of Science during 2014–2024, dated May 20, 2024. c) Network map for critical studies on high‐voltage polymer and quasi‐solid‐state lithium batteries, according to Web of Science during 2014–2024, dated May 20, 2024.

Goodenough et al. provided a schematic illustrating electrode Fermi energy levels and electrolyte electrochemical windows in lithium batteries. The *µA* and *µ*_C_ represent electrochemical potentials at the anode and cathode, respectively. VOC is the open‐circuit voltage of a battery, and the energy separation (*E*_g_) between the lowest unoccupied molecular orbital (LUMO) and the highest occupied molecular orbital (HOMO) of the electrolyte provides the “window” for electrolyte stability. A molecule with a higher HOMO energy implies that it is easier to lose electrons and undergo oxidation. A molecule with a lower LUMO energy indicates that it is easier to accept electrons and undergo reduction. When the Fermi energy level of the anode is higher than the LUMO energy of the electrolyte, electrons will transfer from the anode to the electrolyte, reducing the electrolyte. When the Fermi energy level of the cathode is lower than the HOMO energy of the electrolyte, electrons will migrate from the electrolyte to the cathode, oxidizing the electrolyte. The HOMO and LUMO levels of each molecule can be calculated. By comparing these levels, we can roughly judge which component will react first.^[^ ^10^ ^]^ However, HOMO and LUMO are concepts derived from approximated electronic structure theory when investigating the electronic properties of isolated molecules. Their energy levels do not directly indicate the species participating in redox reactions. Additionally, other molecules and intermolecular interactions can also significantly affect the redox potentials of the electrolyte. Peljo et al. further provided a thermodynamic representation for the electrochemical stability of the electrolyte, based on redox potentials and the Fermi level of electrons in solution (**Figure** **2**a).^[^ ^11^ ^]^ In the past ten years, as shown in Figure 2b, research on high‐voltage polymer and GPE‐based lithium batteries has undergone remarkable development, propelled by the surge in demand for portable electronic devices, electric vehicles, and energy storage systems. As depicted in Figure 2c, a total of 736 studies focusing on high‐voltage polymer and quasi‐solid‐state electrolytes have been further clustered. With the growing demand for high‐energy‐density lithium batteries, increasing attention has been devoted to capacity retention and practical application, which are determined by a robust electrolyte/electrode interphase, an extended electrochemical stability window, adequate interfacial contact, and fast Li⁺ transport across a wide temperature range. Broadening the electrochemical stability window of GPE to match high‐voltage cathodes and promoting Li^+^ transport kinetics are of great significance for realizing high‐energy‐density gel‐polymer‐state lithium batteries with enhanced operability across a wide temperature range.

### 1.2 Cathode Electrolyte Interphase: Formation Mechanism and Role in Electrochemical Stability

**Figure 3:**
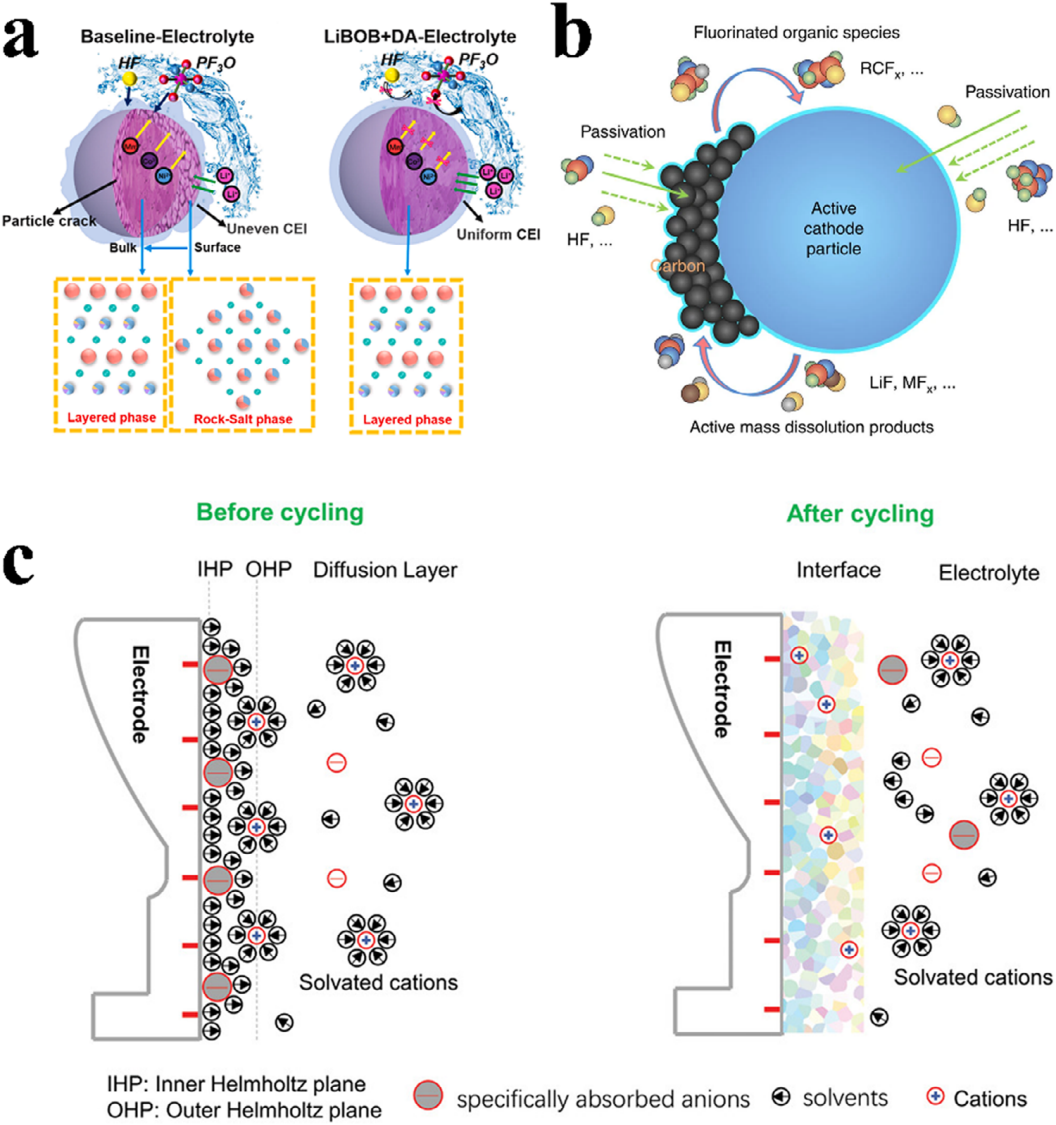
a) The function of the CEI. Reproduced with permission.^[^ ^13c^ ^]^ Copyright 2021, Elsevier. b) Schematic illustrating the spontaneously formed CEI functioning as a passivating film that suppresses side reactions under electrochemical cycling. Reproduced with permission.^[^ ^14^ ^]^ Copyright 2017, Springer Nature. c) Schematic diagram of the interface formation before and after cycling. Reproduced with permission.^[^ ^15^ ^]^ Copyright 2020, Wiley.

Although substantial research has been dedicated to developing electrolytes with inherent stability, it is important to recognize that in real‐world scenarios, electrolytes may still succumb to oxidative breakdown due to their insufficient stability or the catalytic activity of positive electrode materials. This underscores the necessity of establishing a protective barrier, known as the cathode‐electrolyte interphase (CEI), to impede electron transfer from the electrolyte to the cathode.^[^ ^12^ ^]^ The CEI can passivate the cathode surface and prevent direct contact with the electrolyte, thereby playing a crucial role in determining the electrochemical performance of lithium batteries, including their efficiency, cycle life, safety, and irreversible capacity loss (**Figure** **3**a).^[^ ^13^ ^]^ An ideal CEI with chemically, electrochemically, and mechanically stable properties can prevent parasitic reactions between the cathode and the electrolyte, thereby ensuring excellent cycling stability (Figure 3b).^[^ ^14^ ^]^ The initial specific adsorption on the electrode acts as a pioneer, determining the initial structure and chemical composition of the CEI. Meanwhile, the solvated coordination structure functions as a nutrient, maintaining and repairing the interface during cycling (Figure 3c).^[^ ^15^ ^]^ Based on the possible formation mechanisms, many efforts have been devoted to regulating the structure and composition of the CEI via solvation‐tailored strategies, additive engineering strategies, and regulating the electrical double layer (EDL) to achieve high capacity, fast rate capability, and long service life.

In this review, we summarize the design strategies of electrolytes used in high‐voltage lithium batteries, which can be categorized into the following groups: 1) enhancing the intrinsic chemical and electrochemical stability of the electrolyte, and 2) suppressing detrimental reactions between the electrolyte and the positive electrode.

### 1.3 Li^+^ Transport Tinetics: A Key Factor for Rate Capability and Low‐Temperature Performance

It is necessary to develop fast‐charging technology capable of reaching an 80% state of charge within 10–15 min, driven by the increasing population of electric vehicles.^[^ ^16^ ^]^ Ion conductivity stands as a pivotal property of electrolytes, significantly influencing the power output of cells. Given that anions generally do not participate in desired reactions in typical lithium batteries, the Li^+^ transference number ($t_{Li^{+}}$) is introduced to individually quantify the fraction of Li⁺ ions contributing to ion conductivity and thus useful to cell chemistry. The $t_{Li^{+}}$ deserves equal importance to ion conductivity, given that lithium is the sole active element in cells. When there is only one univalent counterion besides Li⁺ ions in the dilute limit, the $t_{Li^{+}}$ can be formulated as follows:^[^ ^17^ ^]^

(1) $$t_{Li^{+}}=\frac{I^{S}R_{b}^{s}\left(\Delta V-I^{0}R_{i}^{0}\right)}{I^{0}R_{b}^{0}\left(\Delta V-I^{s}R_{i}^{s}\right)}$$

where *I* ^0^, $R_{b}^{0}$, and $R_{i}^{0}\;$ represent the initial current, bulk resistance of the electrolyte, and interfacial resistance, respectively. *I^s^*, $R_{b}^{s}$, and $R_{i}^{s}$ stand for the steady‐state current, bulk resistance of the electrolyte, and interfacial resistance, respectively.

In traditional electrolytes, the higher charge density of Li^+^ makes them preferentially solvate with solvent molecules rather than anions, causing the effective radius of Li^+^ to increase significantly after coordinating with several oriented solvent molecules. Consequently, the mobility of the resulting Li⁺ solvation shell is lower than that of anions. For different GPEs, the structure of the polymer host, Li‐salt, plasticizer, and additive together affect the ion conductivity and $t_{Li^{+}}$. The value range of the ion transference number is from zero to one. The $t_{Li^{+}}$ of typical GPEs is only about 0.2–0.4. Various strategies, including incorporating plasticizers, increasing Li‐salt content, and designing polymer chains with functional groups, have been proposed to address these limitations. In this regard, we discuss factors affecting ion conduction and $t_{Li^{+}}$ in GPEs and highlight strategies to improve ion conductivity and $t_{Li^{+}}$ at the molecular level to reduce concentration polarization during the charge and discharge process and increase power density.

**Figure 4:**
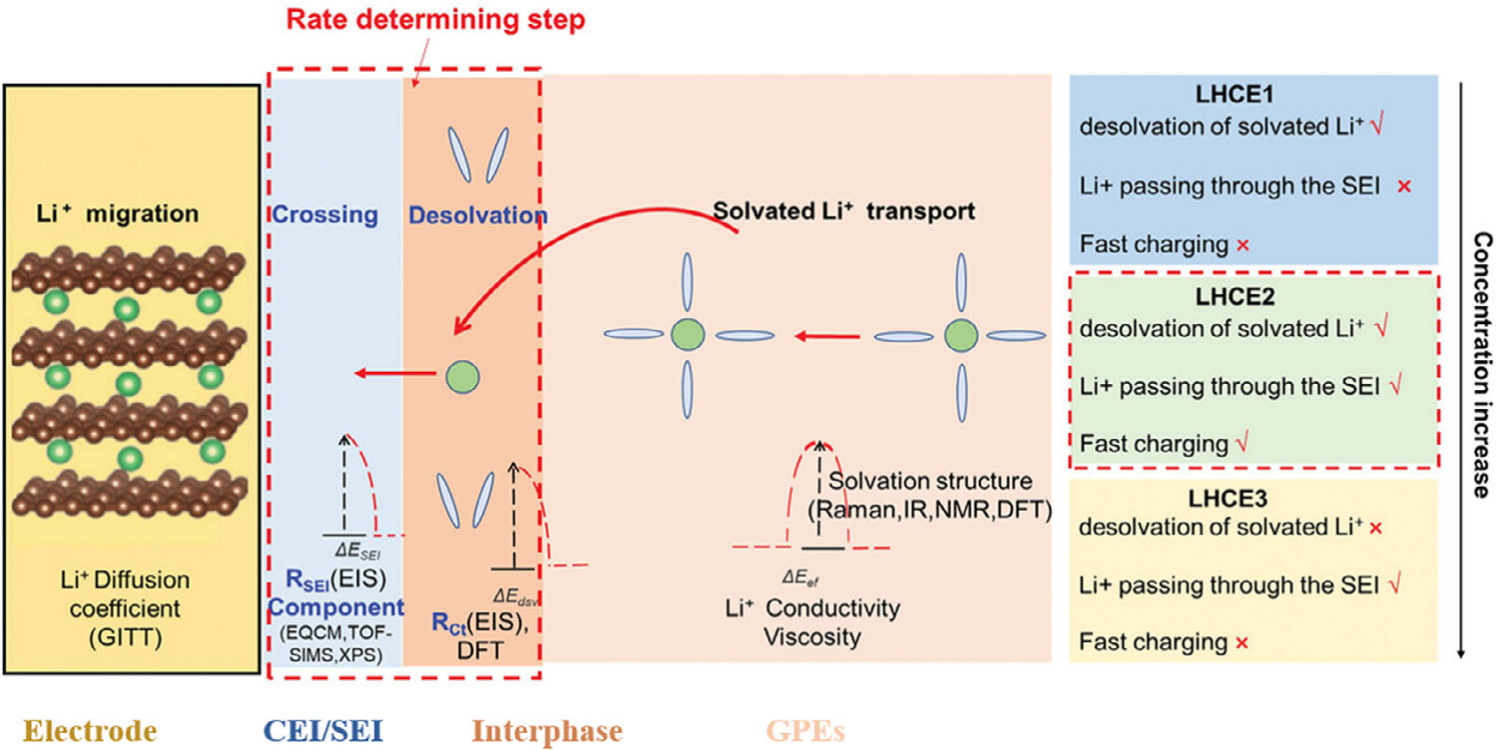
Schematic illustration of Li⁺ transfer in gel polymer electrolyte (GPE)‐based batteries. Reproduced with permission.^[^ ^18^ ^]^ Copyright 2024, Wiley.

For lithium batteries, Li⁺ transport pathways consist of transport in the bulk electrolyte, desolvation at the electrode‐electrolyte interfaces, and migration through the solid electrolyte interphase (SEI) and CEI (**Figure** **4**).^[^ ^18^ ^]^ Previous studies have mainly focused on improving Li^+^ mass transport in electrodes and electrolytes; however, the limitations of charge transfer across electrode‐electrolyte interfaces, including desolvation and migration across CEI/SEI, remain underexplored. Herein, we unravel how molecular regulation and intermolecular interactions reduce Li^+^‐electrolyte binding energy and tune Li^+^ desolvation kinetics in GPEs. Simultaneously, strategies to improve Li^+^ migration through CEI/SEI via molecular‐level interfacial regulation in GPE‐based batteries are summarized. Improving Li^+^ mass transport in electrolytes, desolvation at electrode‐electrolyte interfaces, and charge transfer across CEI/SEI can boost the fast‐charge ability and power density of high‐voltage GPE‐based lithium batteries.

## 2 High‐Voltage Compatibility of GPE

An effective strategy to extend the cycle life of high‐voltage lithium batteries is to enhance the intrinsic chemical and electrochemical stabilities of electrolytes. This approach can significantly suppress parasitic reactions between the cathode and the electrolyte, even when a fresh surface of the positive electrode material is exposed to the electrolyte due to microcracking. Molecular design strategies for intrinsically high‐voltage stable GPEs can be divided into two major categories: 1) increasing the oxidative stability of individual components, including the solvent, Li‐salt, additive, and polymer matrix; and 2) enhancing the intermolecular interactions between the polymer matrix, Li‐salt, solvent, additive, and other components within the GPE.

### 2.1 Molecular Design Strategies for Intrinsically Stable High‐Voltage GPE

The design of the polymer matrix, solvents, and Li‐salts can improve the oxidative stability of GPE by lowering the HOMO level of molecules. Efforts to enhance the intrinsic anodic stability of electrolytes can be divided into the following categories: 1) Substituting a hydrogen atom in a molecule with a high‐electronegativity atom, such as P, S, N, or F. 2) Introducing functional groups with electron‐withdrawing effects, such as –SN, –CN, or –NO₂. 3) Optimizing molecular structures, including extending the conjugated system or increasing the rigidity of the molecule. In this section, we summarized specific strategies from the perspective of the polymer matrix, solvent, and Li salts.

#### 2.1.1 Molecular Chain Design of Polymer Matrix

Since the discovery of GPE in 1975, various polymer hosts have been developed.^[^ ^19^ ^]^ Existing research can be divided into five main categories based on functional units: ether‐based GPE, nitrile‐based GPE, siloxane‐based GPE, carbonate‐based GPE, and fluorine‐based GPE.^[^ ^20^ ^]^ Polyethylene oxide (PEO), a typical polyether chain, is a good complexing agent for Li^+^ ions. However, PEO exhibits a strong electron‐donating ability, as predicted by density functional theory calculations, indicating severe decomposition of PEO under high voltage. Similarly, polycarbonate‐based polymers can coordinate with Li^+^ ions through carbonyl oxygens and exhibit similar properties to some extent. Benefiting from the decrease in HOMO energies, the antioxidative ability of polycarbonate‐based polymers has been enhanced compared to that of PEO. Electron‐withdrawing groups can endow polymer backbones with high‐voltage stability by reducing the electron density of neighboring groups, thereby enhancing the overall oxidation resistance of polyethers and polycarbonates. Low electron density is often associated with better oxidative stability.^[^ ^21^ ^]^ As shown in **Table** **1**, polymers with electron‐withdrawing groups exhibit a high electrochemical stability window (>4.9 V). Therefore, cross‐linking or grafting fluorine/nitrile/sulfone/cationic‐based functional groups is considered an effective approach to enhance the oxidative stability of the polymer chains.

**Table 1:** Chemical structure of some representative GPE for high‐voltage lithium batteries.

Strategy	Solution	Chemical structure	Li salts	Electrochemical window	Refs.
In situ polymerization	G4	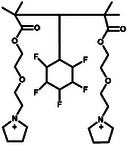	LiTFSI	5.0 V	[22]
In situ polymerization	Pyr_14_TFSI	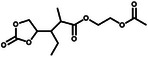	LiTFSI	5.2 V	[23]
In situ polymerization	SN	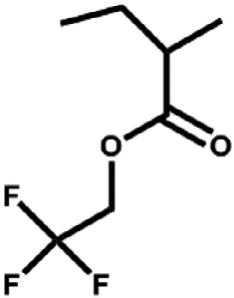	LiTFSI	5.5 V	[24]
In situ polymerization	NML	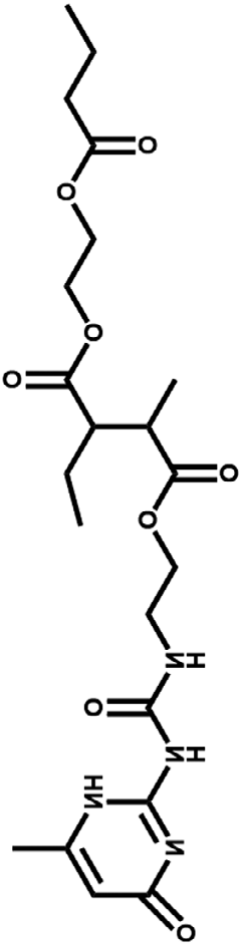	LiTFSI	5.2 V	[25]
Casting	PP_12_FSI	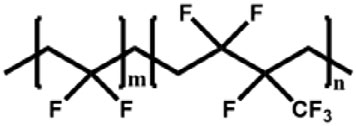	LiFSI	5.0 V	[26]
Casting	NMP	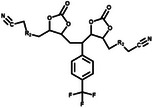	LiTFSI	4.9 V	[27]
Casting	[EMIM][TFSI]	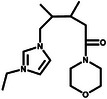	LiFSI	4.95 V	[28]

**Figure 5:**
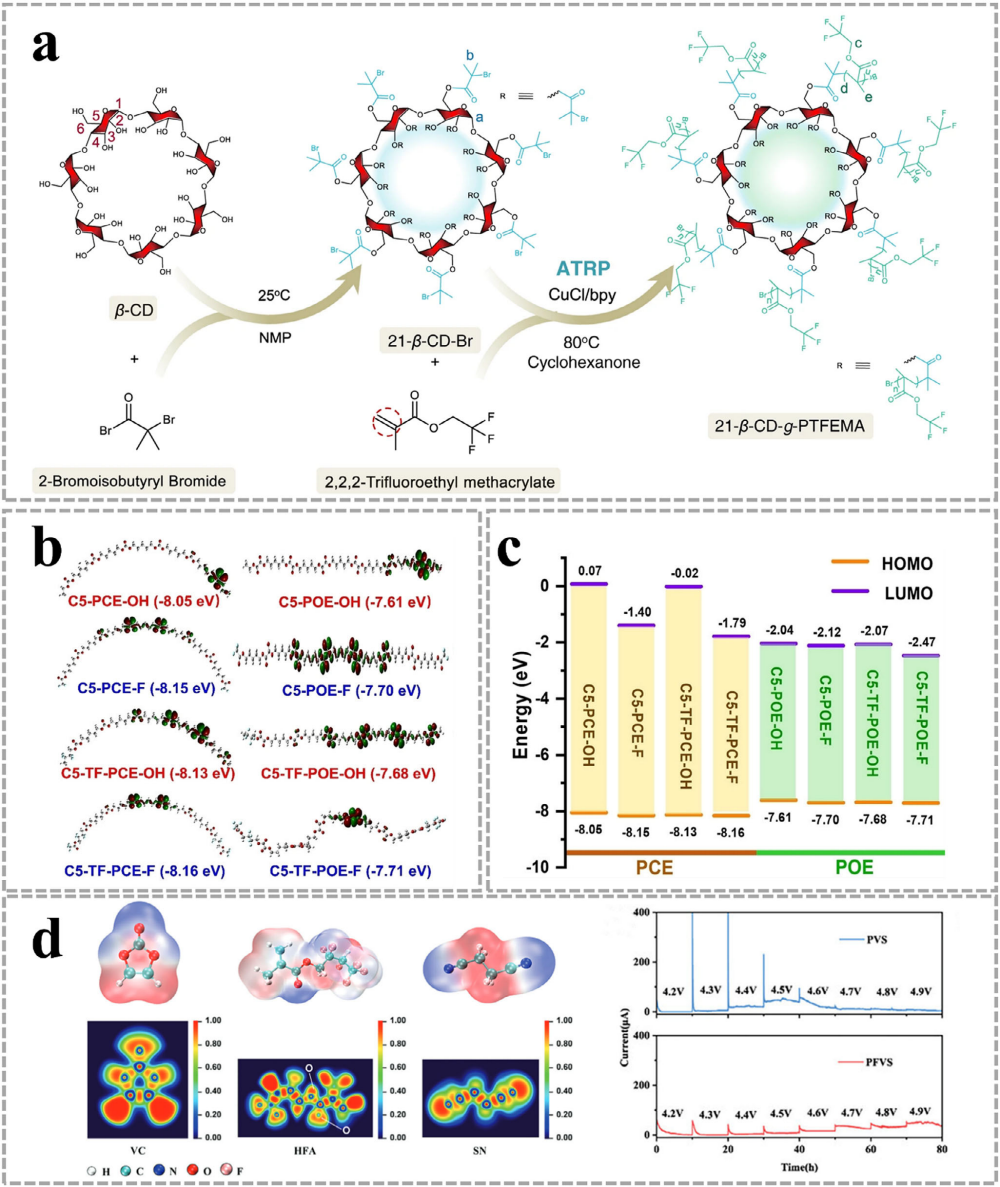
a) Detailed synthetic scheme of 21‐β‐CD‐PTFEMA. Reproduced with permission.^[^ ^29^ ^]^ Copyright 2022, Springer Nature. b) The HOMO molecular orbitals of the hexamers. Reproduced with permission.^[^ ^30^ ^]^ Copyright 2023, Wiley. c) Theoretical calculation of HOMO and LUMO energies of different polycarbonate (PCE) and polyoxalate (POE). Reproduced with permission.^[^ ^30^ ^]^ Copyright 2023, Wiley. d) Electron localization function (ELF) of vinylene carbonate (VC), 2,2,3,4,4,4‐hexafluorobutyl methacrylate (HFA), and succinonitrile (SN), and electrochemical floating analysis of cells assembled with an NCM811 cathode for PVS and PFVS. Reproduced with permission.^[^ ^31^ ^]^ Copyright 2024, Wiley.

Well‐defined 21‐arm fluoropolymers with a postmodified β‐cyclodextrin core and 21 poly(2,2,2‐trifluoroethyl methacrylate) arms (21‐β‐CD‐PTFEMA) were synthesized through atom transfer radical polymerization (**Figure** **5**a). This polymer significantly improves the electrochemical window stability and the Li^+^ transference number.^[^ ^29^ ^]^ The oxidative stability of the ester backbone can be improved by the introduction of terminal trifluoroacetyl units, and the HOMO electrons of the polymer shift to the middle of the main chain, which protects the polymer from chain decomposition reactions (Figure 5b,c).^[^ ^30^ ^]^ A cross‐linked polymer was designed by copolymerization of 2,2,3,4,4,4‐hexafluorobutyl methacrylate (HFA) mixed with vinylene carbonate (VC) and 1 mol% poly(ethylene glycol) diacrylate (PEGDA), labeled as PFVS. PVS represents the copolymer of VC and PEGDA. Delocalization of electron density caused by electron‐deficient fluorine moieties results in the reduction of nucleophilicity of oxygen atoms in the electrolyte and the improvement of the oxidation resistance of the polymer backbone (Figure 5d).^[^ ^31^ ^]^ A special molecular‐level designed polymer electrolyte (MDPE) was obtained by copolymerization of vinylidene carbonate and 4‐vinylbenzotrifluoride. Compared with the electrochemical stability window (ESW) of poly(vinylene carbonate), MDPE can reach an ESW of 4.9 V.^[^ ^27^ ^]^ The effect of the polyfluorinated crosslinker on the ESW of the polyfluorinated cross‐linked GPE was also investigated. The GPE exhibited improved electrochemical oxidation resistance owing to the chemical bonds formed after copolymerization and the fluorine atoms, which can significantly reduce the electron density of neighboring atoms. The assembled Li|GPE|LiNi_0.5_Co_0.2_Mn_0.3_O_2_ with a cutoff voltage of 4.5 V delivered a high discharge specific capacity of 164 mAh g^−1^ after 200 cycles and stable cycling performance with a specific capacity of 146 mAh g^−1^ after 200 cycles.^[^ ^9m^ ^]^ In addition, polymer matrices containing nitrile, ester, and anhydride functional groups also show superior thermodynamic stability owing to the electron‐withdrawing effect. An entanglement association polymer electrolyte was developed based on a poly(vinylidene fluoride‐co‐hexafluoropropylene) (PVFH) matrix and a copolymer stabilizer (PVCA) prepared from acrylonitrile, maleic anhydride, and vinylene carbonate. The entanglement structure and combined functional properties impart high ion conductivity, strong antioxidant potential, and excellent mechanical properties.^[^ ^32^ ^]^

Grafting and copolymerization are effective methods for modifying polymer chains with functional groups, thereby endowing polymer segments with new functional properties. The complexity of the experiments varies depending on the specific context. Gel‐state lithium batteries based on the in situ polymerization process will be a more feasible solution, eliminating the need for separating and purifying the polymer. For example, Mi et al.^[^ ^9k^ ^]^ successfully achieved uniform grafting of lithium phenyl phosphate (LPPO) units onto PVDF through a dehydrofluorination reaction followed by a Friedel‐Crafts alkylation. This process produced a branched, multifunctional solid polymer electrolyte suitable for high‐voltage lithium metal batteries (LMBs). Zhu et al.^[^ ^33^ ^]^ introduced a novel main‐chain fluorinated polymer electrolyte, FEOP, synthesized via in situ cationic ring‐opening polymerization. Combining the high oxidative resistance of polytetrafluoroethylene with the lithium metal compatibility of polyether, FEOP achieves an elevated oxidation potential of up to 5.6 V and facilitates an anion‐involved solvation structure, enhancing its performance in high‐voltage applications. In contrast, Wang et al.^[^ ^27^ ^]^ embedded a special functional group (4‐vinylbenzotrifluoride) within a polycarbonate base. The polymer matrix, obtained through copolymerization and purification of vinylidene carbonate and 4‐vinylbenzotrifluoride, interacts with lithium salt anions via hydrogen bonding and the “σ‐hole” effect of the C─F bond, thereby expanding the electrochemical stability window.

Ether‐based polymers are known for their strong ability to dissociate lithium salts and their good chemical and electrochemical stability with lithium metal.^[^ ^34^ ^]^ However, their relatively narrow electrochemical stability windows limit their compatibility with high‐voltage cathodes. DFT calculations have identified several polymers with better oxidative stability than ether‐based ones, such as polycarbonates (e.g., poly(ethylene carbonate), poly(propylene carbonate), and poly(trimethylene carbonate)), polyesters (e.g., polycaprolactone and poly(β‐propiolactone)), and other polymers like poly(acrylonitrile), poly(methyl methacrylate) (PMMA), and poly(vinylidene fluoride).^[^ ^35^ ^]^ Yet, these polymers face two main challenges: lower stability under reducing conditions and reduced ionic conductivity compared to ether‐based electrolytes. These issues can be addressed through polymer blending, structural modifications, and the addition of plasticizers.

#### 2.1.2 Solvent Design

**Figure 6:**
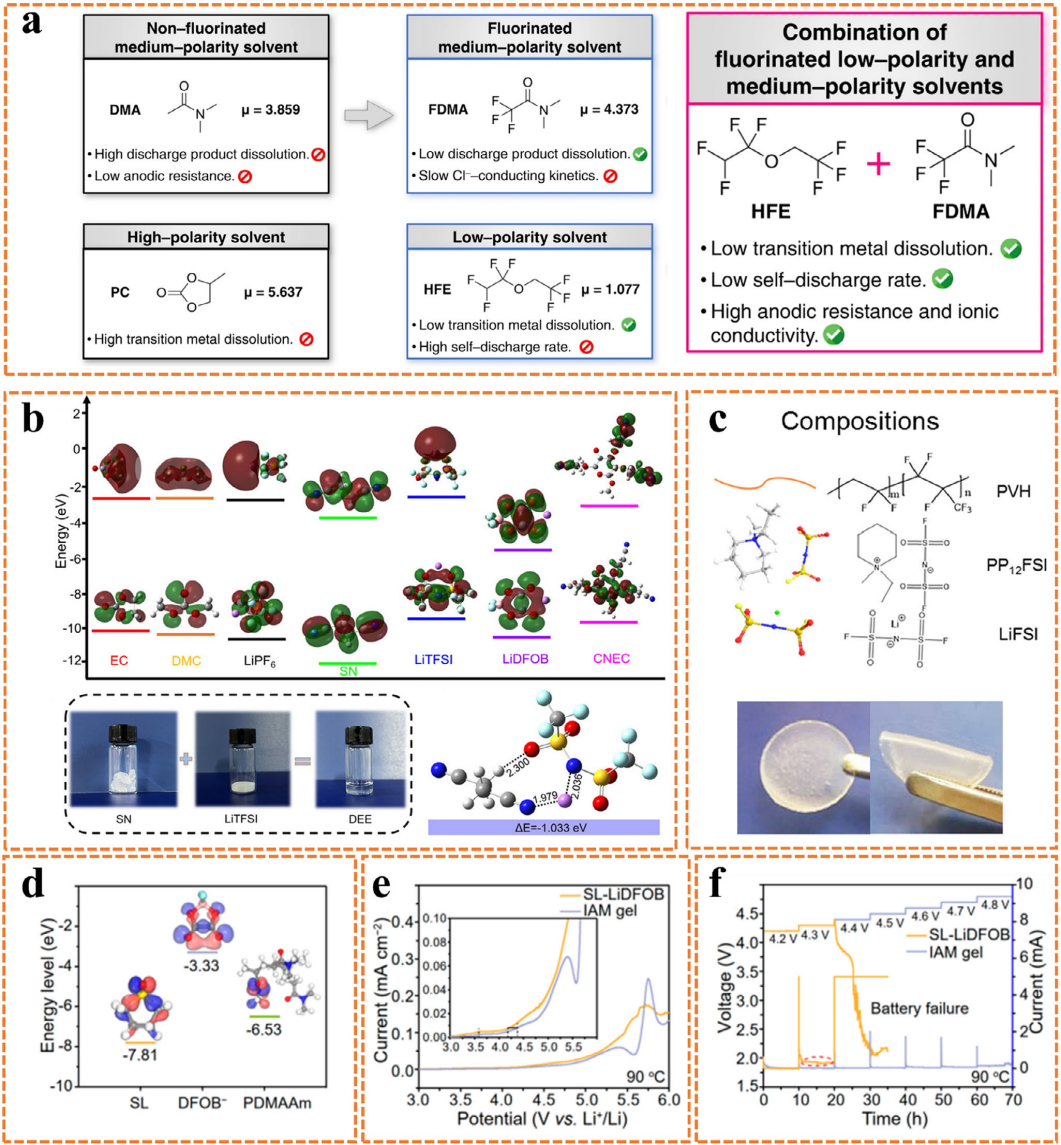
a) Schematic representation of the molecular strategy for the development of binary non‐aqueous electrolyte solvents for liquid Cl‐ion batteries.^[^ ^36e^ ^]^ Copyright 2023, Springer Nature. b) Calculated HOMO and LUMO energy levels and corresponding digital photographs. Reproduced with permission.^[^ ^37^ ^]^ Copyright 2022, Wiley. c) Compositions of the “polymer‐in‐plastic salts” electrolyte. Reproduced with permission.^[^ ^26^ ^]^ Copyright 2020, Elsevier. d) Calculated HOMO energy levels of sulfone, difluoro(oxalato)borate (DFOB^−^), and poly(N,N‐dimethylacrylamide). e) Linear sweep voltammetry (LSV) curves for different electrolytes. f) Electrochemical floating analysis of GPE at 90 °C. Reproduced with permission.^[^ ^36h^ ^]^ Copyright 2020, Elsevier.

Strongly electron‐withdrawing sulfone‐based, nitrile‐based, fluorinated solvents, and ionic liquids exhibit higher oxidation potentials and lower HOMO energies, which follow a similar mechanism to that of the polymer matrix. Thus, fluorinated ethers, fluorinated carbonates, ionic liquids, sulfones, and nitriles with better oxidative stability are considered suitable for high‐energy lithium batteries to achieve desirable cycle stability.^[^ ^26^, ^36^ ^]^ Typical components for fluorinated ether and carbonate electrolytes include fluoroethylene carbonate, 2,2,2‐trifluoroethylmethyl carbonate, 1,1,2,3,3,3‐hexafluoropropyl‐2,2,2‐trifluoroethylether, and fluoromethyl 1,1,1,3,3,3‐hexafluoroisopropyl ether, among others. The introduction of 1,1,2,2‐tetrafluoroethyl‐2,2,2‐trifluoroethyl ether (HFE) to a 1 M tributyl methyl ammonium chloride salt in a 2,2,2‐trifluoro‐N,N‐dimethylacetamide electrolyte can effectively extend electrochemical windows (**Figure** **6**a).^[^ ^36e^ ^]^ Cyanoethyl cellulose and organic plastic salts are candidates for high‐energy lithium batteries due to their appealing electrochemical stability (Figure 6b,c).^[^ ^26^, ^37^ ^]^ Combining a thermally stable sulfone‐based electrolyte with an N,N‐dimethylacrylamide monomer, the prepared GPE maintains high‐voltage stability above 4.3 V at 90 °C (Figure 6d).^[^ ^36h^ ^]^ Additionally, SN, which has a lower HOMO than those of most solvents and Li‐salts, demonstrates high oxidative stability and is expected to protect the electrolyte from continuous electrochemical oxidation decomposition during high‐voltage operations.^[^ ^37^ ^]^ Sulfone‐based and nitrile‐based solvents have poor viscosities and are incompatible with lithium metal anodes. Combining them with other solvents, altering functional groups, or using them as additives can compensate for these drawbacks. Fluorinated solvents have been formulated to enhance oxidative stability and non‐flammability by incorporating electron‐withdrawing ‐CF^n^ groups.^[^ ^38^ ^]^ However, these solvents face significant challenges due to their high viscosity and low ionic conductivity. To address these issues, recent advancements have led to the development of various co‐solvent systems, such as ether solvents, ester solvents, and ionic liquids, which not only mitigate these drawbacks but also improve the rate capability and cyclability of the electrolytes.^[^ ^39^ ^]^

#### 2.1.3 Molecular Design of Li‐Salts

LiPF_6_, featuring a high dielectric constant, low viscosity, and other benefits, is the most popular lithium salt at the moment. However, LiPF_6_ has low thermal stability and can form hazardous HF when it reacts with trace amounts of water in the electrolyte, corroding the CEI and leading to instability in the cathode structure.^[^ ^40^ ^]^ Especially, its performance under high‐voltage conditions is insufficient for practical applications. Thus, it is urgent to develop novel lithium salts with good thermal and chemical stabilities. This section summarizes the effects of high‐voltage lithium salts on battery performance.

**Figure 7:**
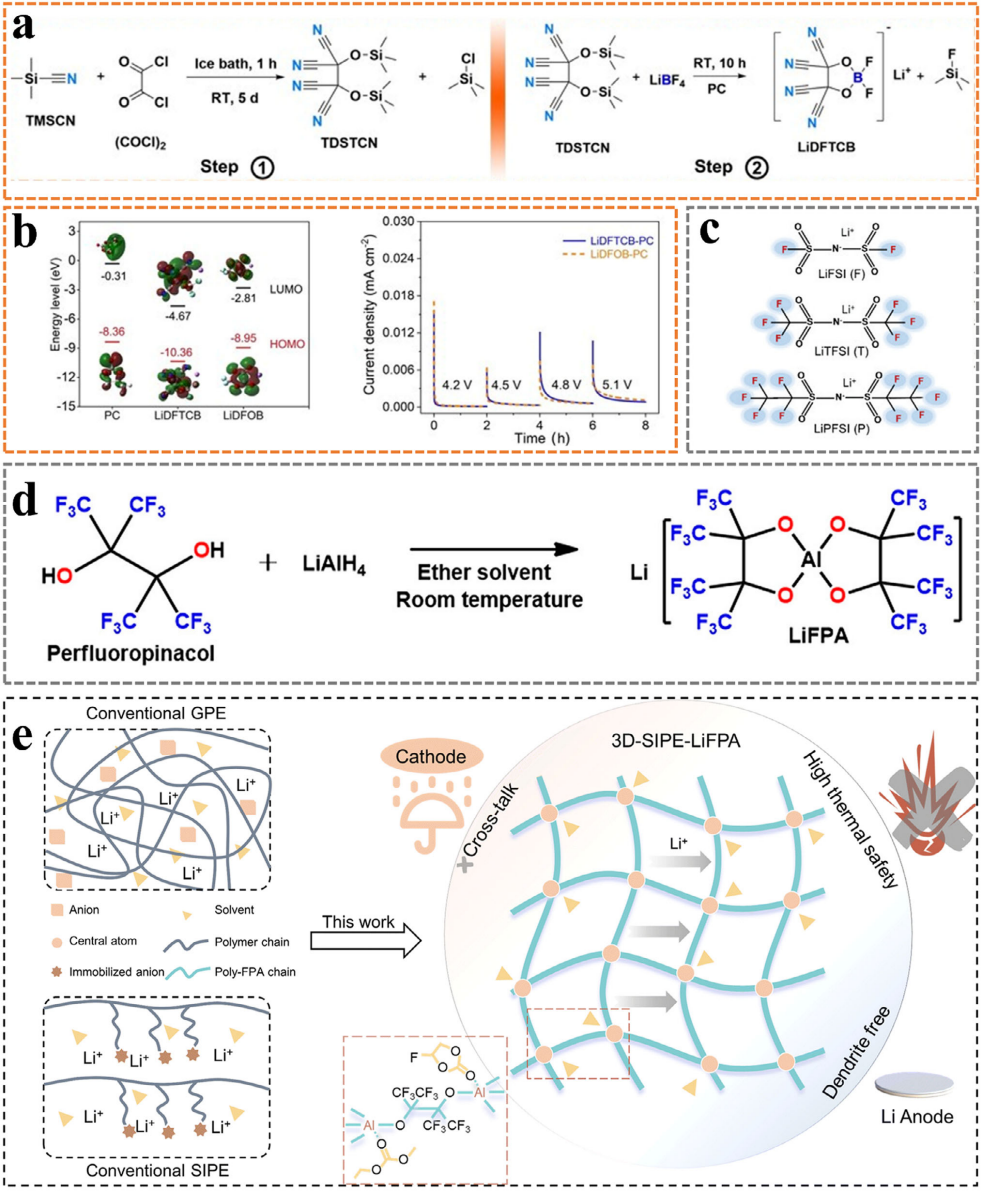
a) Synthetic processes of LiDFTCB. b) HOMO and LUMO energy of propylene carbonate (PC), LiDFOB, and LiDFTCB, and chronoamperometry profiles of Li||Al cells in LiDFTCB‐PC and LiDFOB‐PC electrolytes. Reproduced with permission.^[^ ^42a^ ^]^ Copyright 2023, Wiley. c) Comparison of chemical structures for F‐based Li‐salts. Reproduced with permission.^[^ ^45^ ^]^ Copyright 2023, Wiley. d) Synthetic route of LiFPA. Reproduced with permission.^[^ ^46^ ^]^ Copyright 2022, American Chemical Society. e) Conceptual sketch of conventional GPE, conventional GPE, and as‐constructed 3D‐GPE‐LiFPA. Reproduced with permission.^[^ ^47^ ^]^ Copyright 2023, RSC Publishing.

Borate‐based Li‐salts might replace LiPF_6_ in several applications because of their low production costs and thermal stabilities. The decomposition of lithium bis(oxalato)borate (LiBOB) generates a borate‐containing layer on the surface of cathode particles, suppressing parasitic reactions between the electrolyte and the cathode.^[^ ^41^ ^]^ Lithium difluoro(oxalato)borate (LiDFOB), in combination with advantageous molecular moieties of LiBOB and lithium tetrafluoroborate (LiBF_4_), has become the most promising lithium salt in the field of power batteries. However, several drawbacks have restricted the wide‐scale use of borate‐based Li‐salts, including water sensitivity, difficult purification processes, and low solubility. Introducing strongly electron‐withdrawing cyano and fluorine groups could address the above issues.^[^ ^42^ ^]^ Lithium difluoro(1,2‐dihydroxyethane‐1,1,2,2‐tetracarbonitrile) borate (LiDFTCB) (**Figure** **7**a), a cyano‐functionalized lithium borate salt, shows superior thermal and electrochemical stability under high temperatures and voltages.^[^ ^42a^ ^]^ Lithium perfluoropinacolatoborate (LiFPB), comprising highly fluorinated and borate functional groups, was synthesized as a highly oxidative‐resistant salt to improve the cycle lifespan and safety of practical lithium batteries .^[^ ^42^ ^b]^

Imide‐based Li‐salts have received significant attention due to their high ionic conductivity, thermal stability, and good compatibility with lithium metal. However, imide‐based Li‐salts are corrosive toward Al current collectors at low potentials (> 3.8 V vs Li^+^/Li), excluding their application in 4‐V‐class lithium batteries.^[^ ^43^ ^]^ Hydrogenated sulfonimide salts were designed to remarkably suppress the anodic dissolution of Al current collectors under high potentials and significantly improve the cycling performance of Li||Li(Ni_1/3_Mn_1/3_Co_1/3_)O_2_ cells.^[^ ^44^ ^]^ To investigate the effects of molecular size, fluorine content, and chemical structures (F‐connecting bonds) of Li‐salts on CEI characteristics, three fluorine‐based Li‐salts were selected to prepare solid polymer electrolytes with PEO and polyimide. The HOMO energy levels of lithium bis(pentafluoroethanesulfonyl)imide (LiPFSI) and lithium bis (trifluoromethylsulfonimide) (LiTFSI) are lower than that of lithium bis(fluorosulfonyl)imide (LiFSI), indicating their higher antioxidative ability derived from the strong electron withdrawing effect of −CF_3_ (Figure 7c).^[^ ^45^ ^]^

Other novel Li‐salts also offer superior application potential. A highly fluorinated Al‐centered lithium perfluoropinacolatoaluminate (LiFPA) was innovatively synthesized, enabling high‐loading and high‐voltage lithium batteries with good cycling performance (Figure 7d).^[^ ^46^ ^]^ The use of LiFPA as a monomer for constructing GPE was unprecedentedly proposed (Figure 7e).^[^ ^47^ ^]^ The as‐constructed GPE, enabled by thermal‐induced one‐step in situ polymerization, facilitated a practical Li||NCM811 cell with a long cycle life and thermal stability due to its high Li^+^ transference number and robust CEI/SEI layers. In addition to the ones and analogues mentioned above, lithium 2‐trifluoromethyl‐4,5‐dicyanoimidazole and lithium trifluoromethyl benzimidazole also offer superior application potential, functioning as water purification agents.^[^ ^48^ ^]^ Consequently, selecting proper oxidative‐resistant salts holds great potential for broadening the electrochemical stability window. Simultaneously, the study of the intrinsically oxidative‐resistant mechanisms of Li‐salts and their evaluation in practical cells also needs to be further developed.

### 2.2 Intermolecular Interactions to Enhance High‐Voltage Compatibility

In general, the frontier orbital energy level of polymers, solvents, and Li salts is determined by their intrinsic energy levels.^[^ ^7a^ ^]^ However, intermolecular interactions within GPEs, such as van der Waals forces, hydrogen bonds, ion‐dipole interactions, ion‐ion interactions, dipole–dipole interactions, electrostatic interactions, and π–π interactions, can alter the electron cloud density of GPE components. This, in turn, thermodynamically tunes the HOMO level, raising the energy barriers for parasitic reactions and playing a crucial role in determining high‐voltage stability.^[^ ^9^, ^25^, ^49^ ^]^ In this section, we detailly discuss the effects of intermolecular interactions on encapsulating highly reactive solvents, confining Li salts with higher HOMO energy, and restricting active groups within the polymer matrix.

#### 2.2.1 Encapsulating Solvents by Intermolecular Interactions to Enhance Oxidative Stability

**Figure 8:**
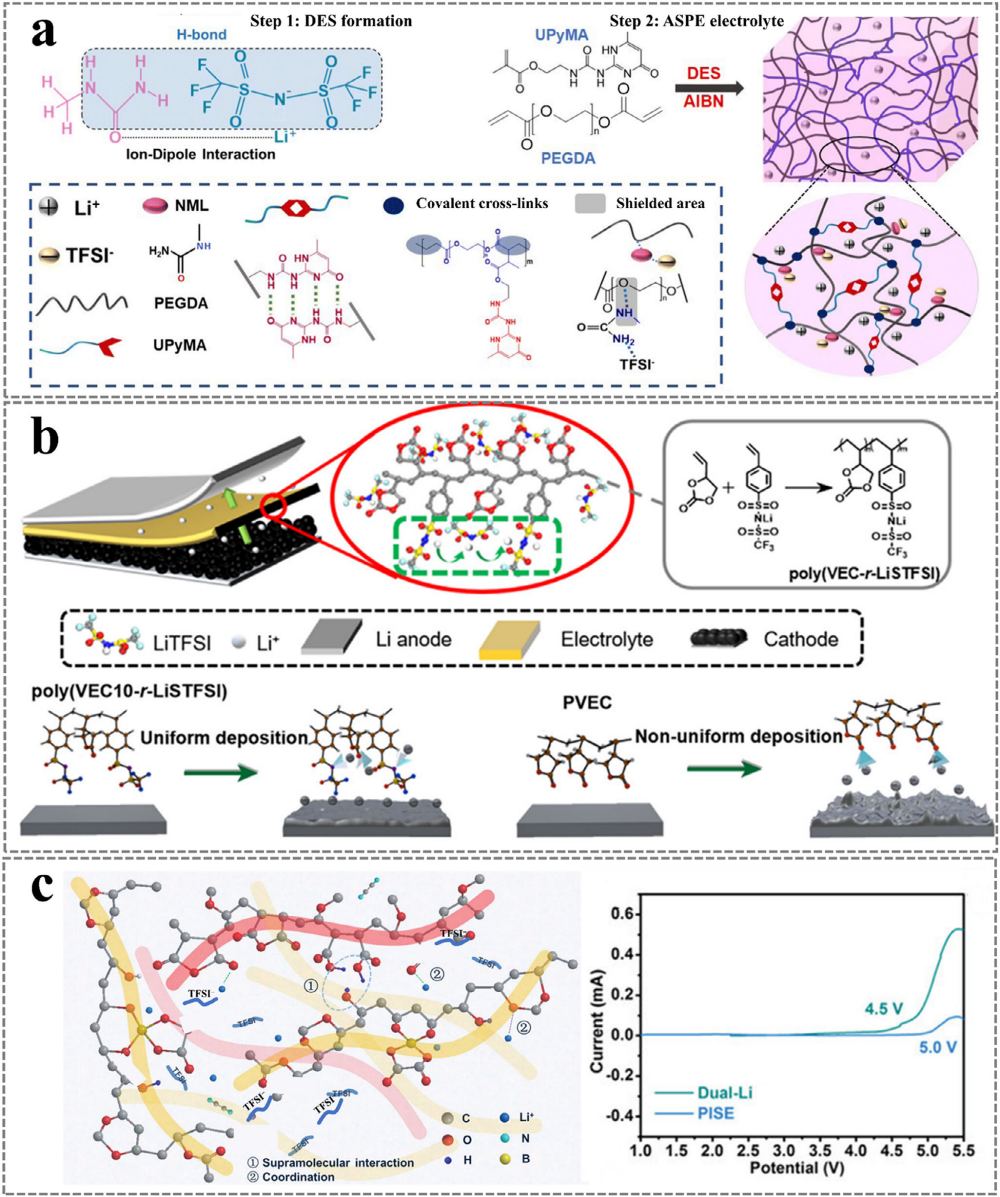
a) Illustrations of DES‐formation reactions and the synthesis process for the polymer electrolyte composed of UPyMA and PEGDA copolymers. Reproduced with permission.^[^ ^25^ ^]^ Copyright 2022, RSC Publishing. b) Illustration of high cationic transport polymer electrolyte (HTPE) design and preparation. Reproduced with permission.^[^ ^53^ ^]^ Copyright 2022, American Chemical Society. c) Sketch of interaction between PME and dual‐Li salts system in PISE. Reproduced with permission.^[^ ^55^ ^]^ Copyright 2021, Wiley.

Electron pair donor solvents with high donor numbers exhibit high solubility for lithium salts, making them ideal choices for fast‐charging lithium batteries. However, these solvents undergo dramatic decomposition on the cathode surface during the charging process, leading to the formation of a high‐resistance CEI film. Electron delocalization, derived from intermolecular electron‐donating and ‐withdrawing interactions between solvents and polymer chains, significantly affects redox properties. Take deep eutectic solvent (DES) electrolytes, and mixtures of LiTFSI and N‐methylacetamide (NMA), as an example. After the polymerization of 2‐(3‐(6‐methyl‐4‐oxo‐1,4‐dihydropyrimidin‐2‐yl)ureido)ethyl methacrylate (UPyMA) and pentaerythritol tetraacrylate (PETEA) monomers in the presence of the DES electrolyte, the self‐healing UPyMA‐PETEA copolymer matrix significantly broadens the electrochemical stability window.^[^ ^50^ ^]^ Similarly, a self‐healing polymer electrolyte was fabricated by in situ copolymerization of UPyMA and PETEA monomers in a deep eutectic solvent (DES)‐based electrolyte containing N‐methylurea (NML) and LiTFSI (**Figure** **8**a).^[^ ^25^ ^]^ The amine group of the NML molecule can interact with the ether group in the polymer chain to form H‐bonds, which can decrease the electron cloud density around ─C─O─C─ and enhance the electrochemical stability of the electrolytes.

Intramolecular interactions between anchored solvents and polymer chains can also effectively raise the energy barriers of parasitic reactions between solvents and cathodes, leading to increased antioxidant capability. For example, the adsorption energy of dimethylformamide (DMF) molecules on grafted PVDF by lithium phenyl phosphate (LPPO) is lower than that on the PVDF chain alone. This strong interaction can gather DMF around the PVDF‐LPPO chain, reducing side reactions between DMF and electrodes.^[^ ^9k^ ^]^

#### 2.2.2 Confined Li‐Salts by Intermolecular Interactions to Boost Oxidative Stability

The electrochemical stability of GPE is also determined by lithium salts, and reducing the anion concentration near the electrode or limiting the movement of anions in GPE can improve electrochemical stability.^[^ ^51^ ^]^ Attaching the anion center to the polymer chain is an effective approach to inhibit the movement of anions. Figure 8b shows that the copolymer composed of imidazolium ionic liquid can effectively immobilize anions through noncovalent interactions with Lewis acidic sites, thereby enhancing the electrochemical window.^[^ ^52^ ^]^ A high cationic transport polymer electrolyte was obtained through in situ polymerization of vinyl ethylene carbonate (VEC) and lithium (4‐styrenesulfonyl) (trifluoromethanesulfonyl)imide (LiSTFSI), which can anchor anions to the polymer backbone and achieve excellent anodic stability.^[^ ^53^ ^]^

Additionally, increasing the concentration of lithium salts can broaden the electrochemical stability of GPE, benefiting from entanglement‐induced rubbery properties.^[^ ^54^ ^]^ A polymer‐in‐salt electrolyte (PISE) with a high lithium salt concentration exceeding 50% was developed via a supramolecular strategy based on poly(methyl vinyl ether‐alt‐maleic anhydride) (PME) and lithiated polyvinyl formal and LiTFSI composite salts. This electrolyte exhibits high ionic conductivity and a wide electrochemical window of over 5 V (Figure 8c).^[^ ^55^ ^]^ First‐principles calculations reveal that the formation of ionic aggregates (AGGs) dramatically improves the oxidative stability of anions because the electrons of anions are donated to Li^+^, and the HOMO energy level of anions is decreased.^[^ ^56^ ^]^

#### 2.2.3 Restricting Active Groups Within The Polymer Matrix to Promote Oxidative Stability

GPEs with dynamic covalent hydrogen bonds and amide bonds in polymer chains exhibit excellent self‐healing ability and maintain good interfacial contact. However, polymer chains containing hydrogen bonds and amide bonds are less resistant to oxidation due to the lower electronegativity of hydrogen atoms. Introducing hydrogen‐bond acceptors into GPEs can modulate the chemical activity of active hydrogen atoms via the formation of hydrogen‐bond intermolecular interactions. These interactions can encapsulate the active hydrogen atoms and increase the energy barrier for chemical reactions with electrodes, thereby suppressing interfacial chemical reactions. Simultaneously, intermolecular electron delocalization between hydrogen‐bond donors and hydrogen‐bond acceptors enables excellent oxidative resistance.

**Figure 9:**
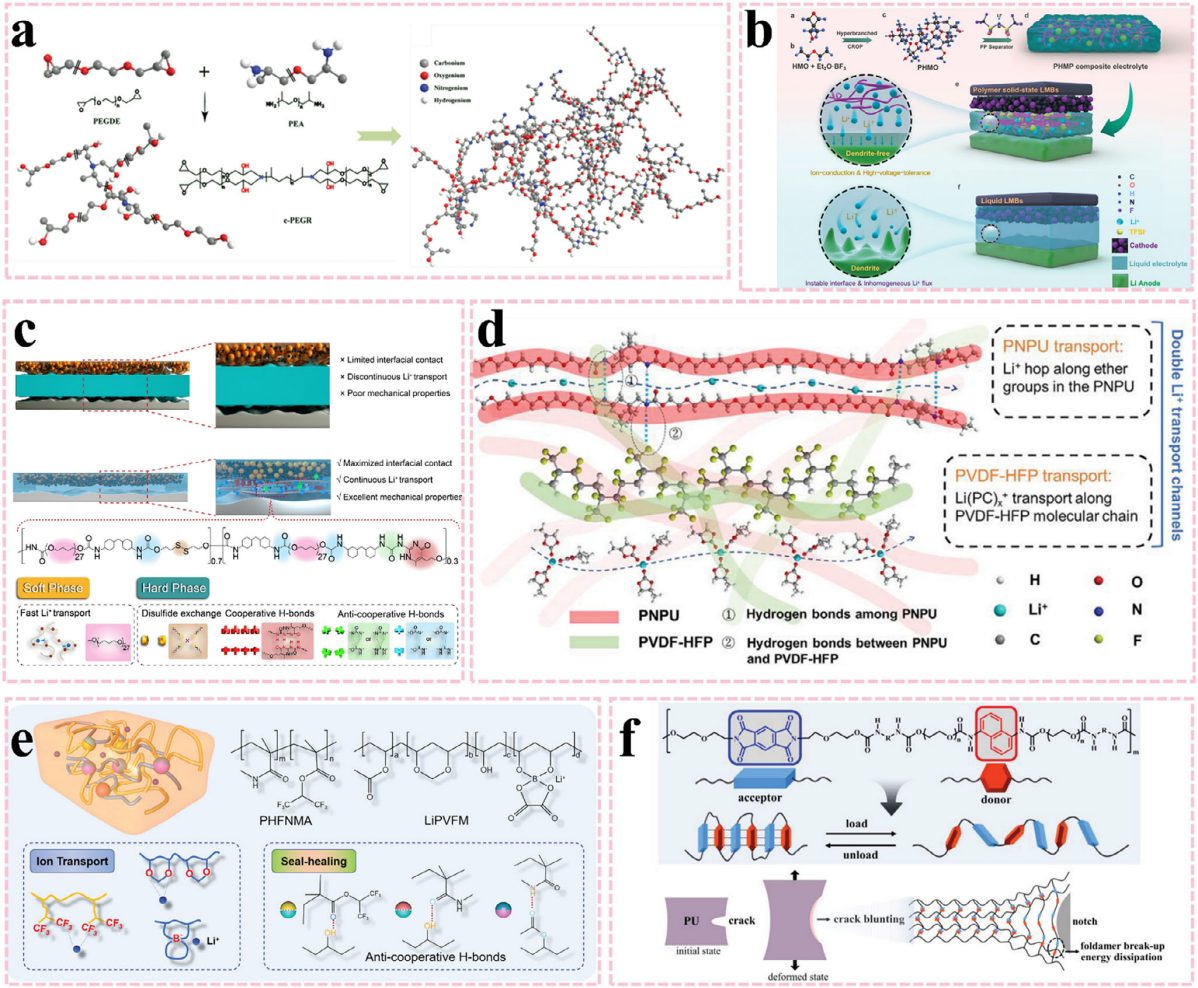
Intermolecular interactions among polymer chains. a) Schematic of the synthesis and structure of c‐PEGR. Reproduced with permission.^[^ ^57^ ^]^ Copyright 2022, Elsevier. b) Synthetic scheme of PHMP composite solid electrolyte. Reproduced with permission.^[^ ^58^ ^]^ Copyright 2021, Wiley. c) The preparation process and molecular structure of the well‐designed DSICE. Reproduced with permission.^[^ ^59^ ^]^ Copyright 2023, Wiley. d) Intermolecular interactions between PVDF‐HFP and PNPU and Li^+^ transport channel of PNPU‐PVDF‐HFP GPE. Reproduced with permission.^[^ ^60^ ^]^ Copyright 2023, Wiley. e) Schematic illustration of the molecular design of dynamic supramolecular SH‐SPE. Reproduced with permission.^[^ ^61^ ^]^ Copyright 2023, Wiley. f) A supramolecular material reinforced by aromatic donor‐acceptor charge‐transfer interactions. Reproduced with permission.^[^ ^62^ ^]^ Copyright 2023, Wiley.

A cross‐linked polyethylene glycol‐based resin (c‐PEGR) was designed through the ring‐opening reaction of epoxy groups and amino groups. The hydroxyl and amino groups, which have inferior oxidation stability, can be restricted within the cross‐linked structure, limiting their freedom of movement. This significantly raises the oxidation potential of c‐PEGR (**Figure** **9**a).^[^ ^57^ ^]^ An ion‐dipole‐reinforced poly‐3‐hydroxymethyl‐3‐methyloxetane has been demonstrated as a novel solid‐state electrolyte and a major alternative to conventional PEO. Its hyperbranched structure restricts the local H⁺ and blocks proton‐induced decomposition at high voltage, contributing to enhanced high‐voltage tolerance (Figure 9b).^[^ ^58^ ^]^ In recent years, supramolecular interactions have been utilized as a driving force to construct intimate electrode/electrolyte interfaces. An integrated cathode/electrolyte for all‐solid‐state lithium batteries was constructed by introducing multiple dynamic bonds, including disulfide bonds (S─S), strong cooperative cross‐linking H‐bonds, and weak anti‐cooperative cross‐linking H‐bonds, into the polymer backbone (Figure 9c).^[^ ^59^ ^]^ A hydrogen bond‐rich network polyurethane (PNPU) was introduced into a PVDF‐HFP polymer matrix to form a self‐healing GPE. The hydrogen bonds and weaker interactions endow the electrolyte membrane with an improved electrochemical window of up to 4.8 V (Figure 9d).^[^ ^60^ ^]^ Poly(hexafluoroisopropyl methacrylate‐co‐N‐methylmethacrylamide) and single‐ion lithiated polyvinyl formal can be incorporated to improve ion conduction and electrochemical window via H‐bond interactions (Figure 9e).^[^ ^61^ ^]^ The cycling stability of solid‐state lithium batteries is closely related to interfacial electrochemical/chemical stability and interfacial contact. Enhancing interfacial electrochemical/chemical stability can suppress interfacial side reactions and lithium dendrite growth, while improving interfacial contact can reduce interfacial impedance. The combined effect of these two factors contributes to the improvement of the cycling performance of solid‐state lithium batteries. To improve the stretchability and self‐healing capability of the electrode/electrolyte interface, a supramolecular polyurethane material reinforced by aromatic charge‐transfer interactions was synthesized (Figure 9f).^[^ ^62^ ^]^ The above investigations indicate that the introduction of molecular interactions can affect the electrochemical window, capacity, and cycling stability. Although significant progress has been made in recent years, the application of supramolecular electrolytes in high‐voltage lithium batteries is still in its infancy due to sluggish Li^+^ transport and electrochemical instability under high voltage. Future efforts may focus on improving fast Li^+^ kinetics and high‐voltage compatibility.

The conventional materials design approach, relying on fundamental laws, computational modeling, and experimentation, struggles when dealing with large datasets. Consequently, there is an urgent need to develop new tools capable of handling extensive data. Artificial intelligence (AI)‐based methodologies have emerged as a promising solution to accelerate materials design and development. For instance, Gao et al. combined machine learning with density functional theory to predict donor numbers for polymer building blocks, enabling the design of polymer chains with optimized donor numbers that effectively modulate the Li⁺ solvation structure and enhance rapid Li⁺ transport kinetics.^[^ ^63^ ^]^ Similarly, Qin et al. demonstrated an AI‐assisted workflow for wide‐temperature electrolyte design through sequential parameterizations and calculations, identifying linear mono‐nitriles as ideal solvents with a broad liquidus range.^[^ ^64^ ^]^ Moving forward, AI‐assisted methods hold the potential to expedite electrolyte design by providing multi‐dimensional guidance and clarifying complex structure‐performance relationships, such as elucidating inter‐ and intramolecular interactions in practical electrolytes.^[^ ^65^ ^]^

In this section, we have delved into the mechanisms of electrolyte regulation at the molecular level, encompassing the design of polymer matrices, the selection of solvents, the utilization of lithium salts, and the establishment of molecular interactions. The development of high‐voltage‐resistant components for GPEs and the introduction of molecular interactions within GPEs emerge as promising directions for engineering GPEs with superior electrochemical performance.

## 3 Molecular Regulation to Enhance Li^+^ Transport Kinetics

Promoting Li^+^ transport kinetics based on GPEs involving solvated Li^+^ transports through bulk GPE, Li^+^ desolvation process at the electrode/electrolyte interface, and Li^+^ migration through CEI/SEI layers, which is crucial for achieving a stable and high‐rate lithium batteries.

### 3.1 Li^+^ Transport Kinetics within GPEs

Understanding the ionic conduction mechanisms is essential for improving Li^+^ transport kinetics within GPEs. This section discusses ionic conduction and strategies to enhance the ionic conductivity and Li^+^ transference number ($t_{Li^{+}}$), defined as the fraction of ionic conductivity imparted by the lithium‐ion.

#### 3.1.1 Ionic Conduction in GPEs

GPEs are complexes formed by reactions of lithium salts with polar or Lewis‐acid‐base active groups of polymer matrices and solvents. The ionic conductivity of GPEs depends heavily on diffusion pathways. A comprehensive understanding of the Li^+^ transport mechanism is of supreme importance for designing high‐performance GPEs with high capacity, fast rate capability, and long service life. The Li^+^ transport pathways in GPEs are closely related to the ability to form homogeneous complexes and the ability of polymers and solvents to dissolve salts and dissociate Li^+^. The solvating capability of the polymer matrix and solvents in electrolytes can be illustrated by the dielectric constant, Gutmann donor number (DN), and relative solvating capability^[^ ^66^ ^]^ Theoretical calculations or simulations, including surface electrostatic potential and binding energy, have also been adopted to investigate the solvating capability of polymer matrices and solvents. Additionally, the dissociation energy of coordinated Li^+^ with solvents or polymer matrices plays a vital role in determining the ion conduction mechanism. The potential Li⁺ transport pathways in bulk GPEs include: 1) Transfer through the polymer matrix, 2) Transfer along the liquid phase, 3) Interconnected Li^+^ conductive channels constructed by the coordination between the polymer matrix and Li^+^ and between the liquid phase and Li⁺ synergistically, 4) Li^+^ transport based on interactions between [solvent‐Li⁺] complexes and polymers, and Li^+^ transport via electron‐delocalization interfaces, which critically depends on Li⁺ affinity and the content of solvents and polymer matrices.

##### Li^+^ Transport Through Polymer Matrix or Liquid Phase

Some solvents are unable to dissolve lithium salts or participate in the Li⁺ solvation structure but can swell the polymer matrix. The Li⁺ transport in the GPE is only correlated with polymer segment motion, as verified by a hybrid GPE consisting of poly(vinyl chloride) (PVC), poly(methyl methacrylate) (PMMA), lithium tetrafluoroborate (LiBF₄), and dibutyl phthalate (DBP).^[^ ^67^ ^]^ The ionic transport can be facilitated by the movement of the main‐chain segments, which can be facilitated through various polymer engineering techniques such as copolymerization, cross‐linking, and grafting. Alternatively, it can be realized by adding plasticizers or increasing the temperature above the glass‐transition temperature. These methods can enhance the segmental movements of the polymer chains, thereby improving the ionic transport properties.^[^ ^68^ ^]^

**Figure 10:**
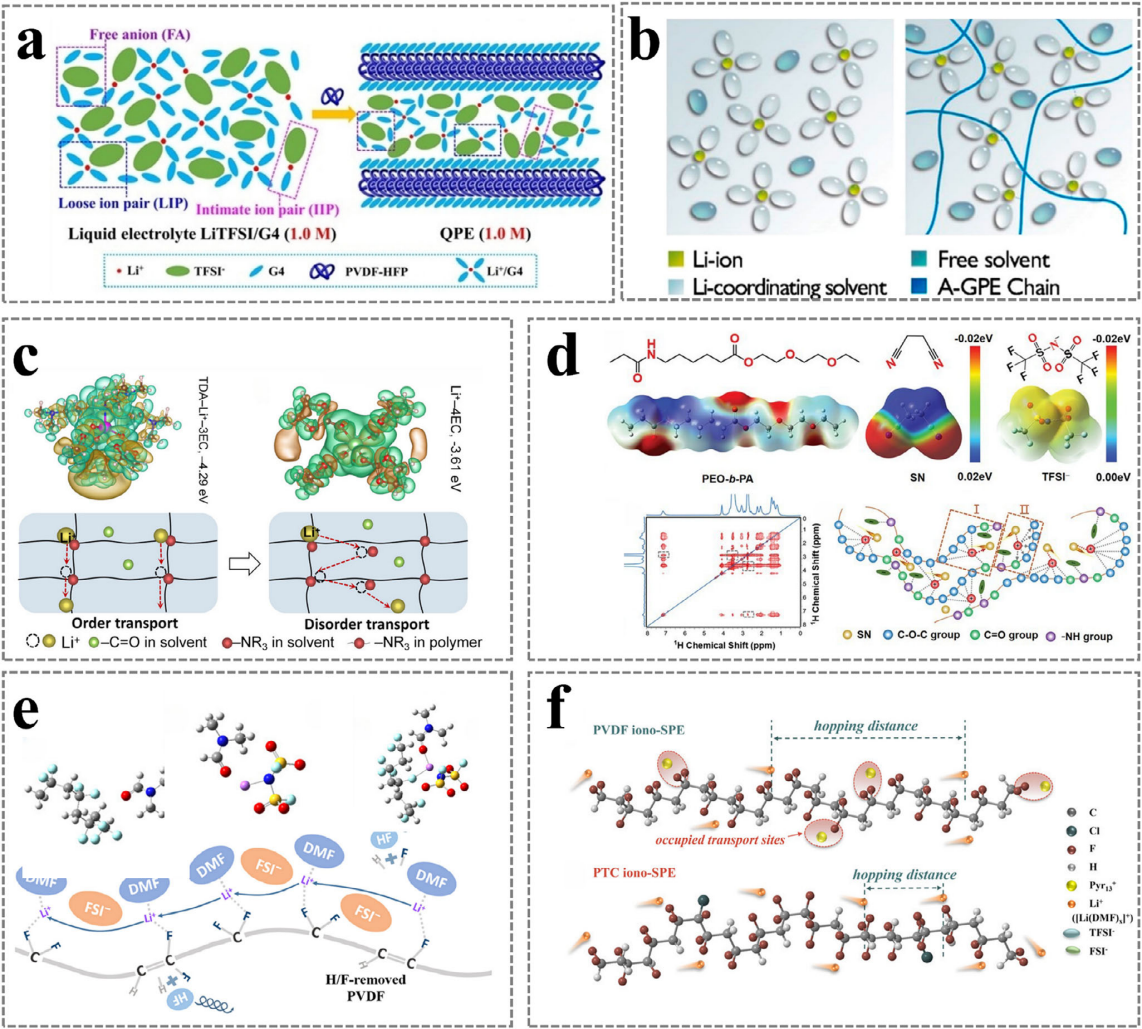
a) Schematic of the solvation model in LiTFSI/G4 and PVDF‐HFP + LiTFSI/G4 GPE. Reproduced with permission.^[^ ^69^ ^]^ Copyright 2021, Wiley. b) Illustration of Li⁺ solvation structures in liquid electrolyte and A‐GPE. Reproduced with permission.^[^ ^71^ ^]^ Copyright 2019, American Chemical Society. c) Binding energy and schematic of Li⁺ transport from order to disorder. Reproduced with permission.^[^ ^73^ ^]^ Copyright 2023, Wiley. d) Electrostatic potential (ESP) comparison of PEO‐b‐PA, SN, and TFSI⁻ anions and diagrams for fast Li⁺ transmission paths. Reproduced with permission.^[^ ^74^ ^]^ Copyright 2023, Wiley. e) The optimized structures of the DMF‐PVDF, DMF‐LiFSI, and PVDF‐DMF‐LiFSI systems and a schematic illustration of the interactions and Li⁺ transport in the “dried” PVDF‐DMF‐LiFSI electrolyte. Reproduced with permission.^[^ ^76^ ^]^ Copyright 2022, Elsevier. f) Schematic diagram of Li⁺ transport along molecular chains in PVDF iono‐SPE and PTC iono‐SPE. Reproduced with permission.^[^ ^77^ ^]^ Copyright 2023, Wiley.

If the polymer matrix cannot dissolve lithium salts or participate directly in the Li⁺ solvation structure and merely serves as an inert mechanical framework, Li⁺ transport occurs predominantly through the liquid phase. A typical PVDF‐HFP‐based model GPE, consisting of PVDF‐HFP, LiTFSI, and tetraethylene glycol dimethyl ether (G4), was analyzed using a multispectral characterization strategy combined with first‐principles calculations. The results revealed that the solvation structure and coordination number of Li⁺ are similar to those in liquid electrolytes, demonstrating that G4 solvents in the GPE dissolve Li salts and swell the polymer matrix (**Figure** **10**a).^[^ ^69^ ^]^ When a liquid electrolyte (LiTFSI in G4) with a high volume content (nearly 60%) is confined within a polyacrylonitrile‐poly(ethylene glycol methyl ether methacrylate) (PAN‐co‐PEGMEMA) polymer matrix, the GPE shows direct coordination of Li⁺ ions with G4, leading to the formation of [Li(G4)]⁺ clusters. Li⁺ transport is independent of polymer segmental motion and is governed solely by [Li(G4)]⁺ clusters.^[^ ^70^ ^]^ For an acrylate‐based GPE (A‐GPE) containing 2 wt.% acrylate monomer and liquid electrolyte (1M LiPF₆ in ethylene carbonate (EC)/ethyl methyl carbonate (EMC) (1/2 v/v)) (Figure 10b), the functional groups of the acrylate‐based polymer matrix can participate in the Li⁺ solvation structure. However, the activation energy for ion conduction in A‐GPE is quite similar to that in liquid electrolytes, indicating that there is no significant change in Li⁺ transport pathways, probably due to the low content of the polymer matrix.^[^ ^71^ ^]^ Enhancing the liquid electrolyte uptake proves effective. Additionally, incorporating functional groups into the polymer matrix can convert the original ion‐insulating polymer into a fast ion conductor. For instance, Dai et al. incorporated trifluoroethylene and chlorofluoroethylene monomers into the PVDF crystals, introducing dipolar defects. This approach transformed the initially ion‐insulating PVDF crystalline phase into a rapid ion conductor.^[^ ^72^ ^]^

##### Li^+^ Synergistic Transport in Interconnected Liquid Phase and Polymer Matrix

Compared with the aforementioned Li⁺ transport pathways through the liquid phase or polymer matrix, the solvation of lithium salts in both the liquid phase and polymer matrix can synergistically construct interconnected Li⁺ transport pathways, enabling Li^+^ to transport through the liquid and swollen, gelled phase, as well as through the segment motion of polymer chains. This multimodal transport mechanism often yields ionic conductivities surpassing those of SPEs and approaching those of liquid electrolytes. This effectively reduces the concentration gradient of Li⁺ ions and improves their uniform distribution at the electrode/electrolyte interface. Benefiting from the higher coordination strength of Li⁺ on the tertiary amine (─NR₃) group of the polymer network than on the carbonyl (C═O) group of the ester solvent, Li⁺ can diffuse orderly and quickly along the ‐NR₃ groups of the polymer, delivering excellent ion conductivity of 3.69 mS cm^−1^ (Figure 10c).^[^ ^73^ ^]^ A polyether‐b‐amide (PEO‐b‐PA) based GPE containing LiTFSI, SN, and a glass fiber mesh was fabricated through a simple solvent‐free method (Figure 10d).^[^ ^74^ ^]^ The strong electron density in the ether and acyl groups (─C─O─C─/─N─H─) and nitrogen atoms of the cyano group contributes to the coordination of PEO‐b‐PA and SN with Li⁺, facilitating Li⁺ transport in the interconnected liquid phase and polymer matrix. An ion‐selective “skin” was constructed via in situ gelation using isocyanoethyl methacrylate‐grafted polyethylenimine (PEI‐IEM) in the electrolyte. Benefiting from the hyper‐branched network structures and abundant polar groups (─N─H─/─C═O─) in this designed skin, the PEI‐IEM GPE achieves excellent regulation of Li⁺ ions. This is attributed to ionic self‐concentrating kinetics, leading to a higher local concentration of Li⁺ ions than in the bulk solution and yielding efficient Li⁺ conduction and dendrite‐free Li deposition on the Li anode.^[^ ^75^ ^]^

##### Li^+^ Transport Based on Interactions Between [Solvent‐Li^+^] Complexes and Polymers

In some GPEs, Li⁺ conductivity is dominated by the polymer matrix and the immediately surrounding solvent, which is different from the previous ion conductivity mode. The change in the conduction model can be ascribed to the unique Li⁺ solvation structure in GPEs: all solvent molecules appear in the form of [solvent‐Li⁺] complexes, and the polymer strongly interacts with these [solvent‐Li⁺] complexes rather than with the naked Li⁺ ions or solvents.

Take a PVDF‐based electrolyte with a low content of solvent as an example (the content of DMF is 15%). PVDF has minimal interaction with Li⁺, while the [DMF‐Li⁺] complex has a strong interaction with PVDF. Figure 10e schematically shows the Li⁺ conduction mechanism in this GPE. All solvent molecules appear in the form of [solvent‐Li⁺] complexes, and the polymer strongly interacts with these [solvent‐Li⁺] complexes rather than with the naked Li⁺ ions or solvents.^[^ ^76^ ^]^ A similar study by Liu's group confirmed that poly(vinylidene fluoride‐trifluoroethylene‐chlorotrifluoroethylene) [P(VDF‐TrFE‐CTFE) (PTC)] is employed as the framework for N‐propyl‐N‐methylpyrrolidinium (Pyr13)‐TFSI to prepare a novel iono‐SPE (Figure 10f).^[^ ^77^ ^]^ PTC, with its moderate local polarity and extremely high dielectric constant, reduces the Li⁺ migration barrier and promotes the dissociation of ion clusters, thereby improving Li⁺ transport efficiently in the polymer phase and inducing a uniform Li⁺ flux.

##### Li^+^ Transport via Electron‐Delocalization Interface

Although fluoroethylene carbonate (FEC) has a high dielectric constant, liquid electrolytes comprising FEC and LiDFOB fail to transport Li⁺ effectively. Previous studies have implied that the relative solvating capability of a solvent decreases with increasing fluorination degree.^[^ ^66^ ^]^ However, in situ polymerized GPEs comprising FEC and carbonate ester segments (F‐GPE) demonstrate superior electrochemical performance. The polymer segments swell with the incorporation of FEC, and an electron‐delocalization interface layer is generated between the abundant electron‐rich groups of FEC and the polymer matrix. This interface acts as an electron‐rich “Milky Way,” facilitating the rapid transfer of Li⁺ by dramatically lowering the diffusion barrier.^[^ ^78^ ^]^

Considering that GPEs possess the cohesive characteristics of solids and the diffusive attributes of liquids, optimizing the components of GPEs including the polymer matrix, salts, encapsulated liquids, and salts‐solvent‐polymer interactions, is crucial for achieving high ionic conductivity. Although necessary, only high ionic conductivity does not guarantee excellent rate performance over a wide temperature range. This is because ionic conductivity results from the collective movement of all ionic species, whereas the faradaic current is primarily balanced by Li^+^ ions rather than their counterions.^[^ ^79^ ^]^ In fact, anions usually have smaller dimensions and higher mobility, enabling them to navigate the electrolyte more rapidly.^[^ ^80^ ^]^ In practical electrolyte systems, the design principles for GPEs focus on increasing the lithium‐ion transference number without substantially sacrificing ionic conductivity.

#### 3.1.2 Strategies to Improve Li^+^ Transference Number

**Figure 11:**
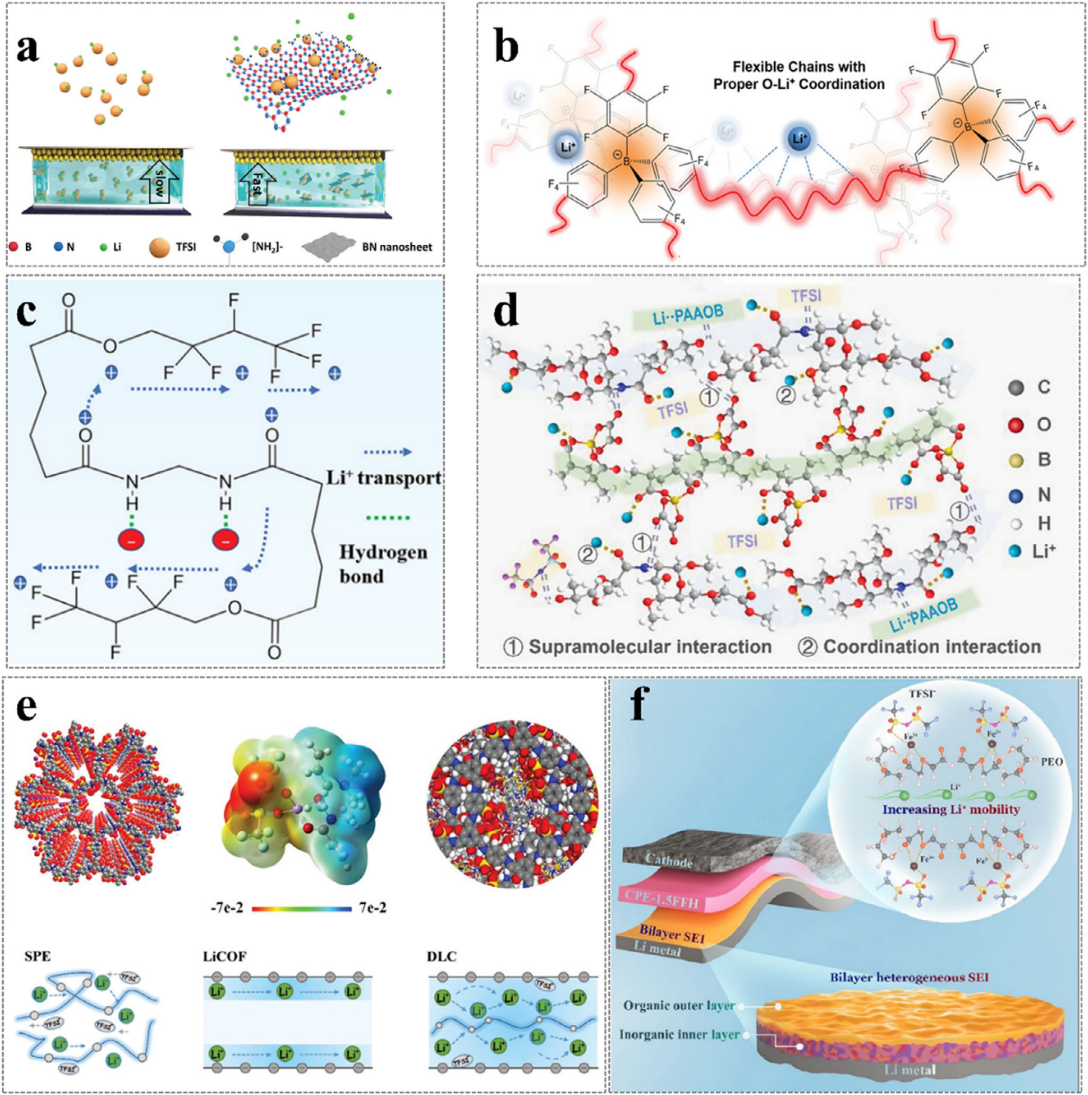
Schematic of mechanisms to enhance $t_{Li^{+}}$. a) Li^+^ conduction in ion GPE without and with 1.5 BN nanosheets. Reproduced with permission.^[^ ^81^ ^]^ Copyright 2020, Wiley. b) Copolymer of LiCTFPB with tetraethylene glycol. Reproduced with permission.^[^ ^84^ ^]^ Copyright 2022, Wiley. c)Hydrogen bond interactions. Reproduced with permission.^[^ ^86a^ ^]^ Copyright 2023, Wiley. d) Diagram of supramolecular interactions between polymer matrix and Li‐salts. Reproduced with permission.^[^ ^89^ ^]^ Copyright 2023, Wiley. e) Illustration of Li^+^ transport modes in conventional solid‐state electrolyte, pristine LiCOF, and liquid electrolyte‐mediated COF electrolyte. Reproduced with permission.^[^ ^90^ ^]^ Copyright 2022, Wiley. f) Weakening Li^+^‐O interactions to elevate Li^+^ mobility. Reproduced with permission.^[^ ^91^ ^]^ Copyright 2023, Elsevier.

Restricting anion movement or improving Li⁺ mobility are primary ways to increase the $t_{Li^{+}}$ of GPEs. Li‐salt concentration and GPE structure significantly influence $\;t_{Li^{+}}$. Introducing Lewis acid groups into GPEs can trap anions and reduce the dissociation energy of Li‐salts, thereby enhancing Li⁺ mobility and slowing down anion movement.^[^ ^81^ ^]^ (**Figure** **11**a) illustrates that Li⁺ mobility in ion gel electrolytes is significantly enhanced because anions are likely anchored by functionalized boron nitride (BN) nanosheets, thus leaving freer Li⁺. Aluminum fluoride, a Lewis acid additive, was introduced into a poly(vinylene carbonate) electrolyte to immobilize TFSI⁻ anions and improve electrochemical properties.^[^ ^82^ ^]^ Copolymerization of Li‐salts and monomers can fix anions to the polymer backbone, restricting anion migration and leading to a high $t_{Li^{+}}$ close to unity.^[^ ^83^ ^]^ Cross‐linking lithium tetrakis(4(chloromethyl)‐2,3,5,6‐tetrafluorophenyl)borate salt (LiCTFPB) with tetraethylene glycol results in the fabrication of an interpenetrating single‐ion network polymer, delivering a superior $t_{Li^{+}}$ of 0.92 (Figure 11b).^[^ ^84^ ^]^ Based on the copolymerization of VEC and lithium 3‐sulfonyl(trifluoromethanesulfonyl)imide propyl methacrylate plasticized by minimal succinonitrile, a novel single‐ion polymer conductor was synthesized. This conductor can immobilize anions to the backbones of polymer chains and enhance $t_{Li^{+}}$ up to 0.93.^[^ ^85^ ^]^ Hydrogen bond interactions between ─NH, ─OH, and ─F groups in the polymer matrix and anions can promote the increase of $t_{Li^{+}}$ by anchoring anions to the polymer framework.^[^ ^86^ ^]^ The abundant acylamino group (O═C─NH) and F groups in the polymer matrix are conducive to dissociating Li‐salts and promoting the improvement of $t_{Li^{+}}$ (Figure 11c).^[^ ^86a^ ^]^ Additionally, electrostatic interactions can further retard anion mobility by electrostatic attraction and facilitate Li⁺ transfer via electrostatic repulsion. An ion gel electrolyte membrane comprising polymerized ionic liquid nanofibers and cross‐linked poly(2,2,2‐trifluoroethyl methacrylate) was reported. The positively charged poly(ionic liquid) (PIL) backbone has a stronger interaction with anions, which can significantly enhance $t_{Li^{+}}$.^[^ ^87^ ^]^ Supramolecular interactions between the polymer matrix and Li‐salts, including hydrogen bonds, Van der Waals forces, and metal‐ligand coordination, are also vital for increase $t_{Li^{+}}$. For example, supramolecular interactions between poly(propylene oxide/2‐(2‐methoxyethoxy)ethyl glycidyl) and fluorinated anion‐based salts can greatly improve $t_{Li^{+}}$, interfacial compatibility, and mechanical stability.^[^ ^88^ ^]^ Strong supramolecular interactions between the polymer skeleton containing polar functional groups (─C═O, ─C─O, ─NH₂, and ─OH) and supramolecular lithium polyacrylic acid oxalate borate enable the electrolyte with excellent ion conductivity and $t_{Li^{+}}$ (Figure 11d).^[^ ^89^ ^]^ Filling GPEs with ionic covalent organic frameworks (COFs) is a practical strategy to promote Li⁺ conductivity. For example, an electrolyte‐mediated single‐Li⁺‐covalent organic framework (COF) (Li‐COF) was fabricated. In situ solidification of a tailored liquid electrolyte can boost the charge‐carrier concentration in the COF channels and decouple Li⁺ from both COF walls and molecular chains (Figure 11e).^[^ ^90^ ^]^

Modulating the coordination environment of Li^+^ can weaken the transfer barrier of Li^+^, leading to higher ionic conductivity and $t_{Li^{+}}$. Figure 11f shows that the coordination competition between Li^+^ and Fe^3+^ with ether oxygen in the PEO chain is the driving force for the reconfiguration of surrounding Li^+^ ions, which releases free Li⁺ ions for superior Li^+^ mobility.^[^ ^91^ ^]^ In poly(4‐acryloylmorpholine) (ACMO) electrolytes, Li⁺ ions tend to bind with carbonyl groups in ACMO and transport slowly along the polymer chains. After copolymerizing ACMO with 1‐vinyl‐3‐ethylimidazolium bis(trifluoromethylsulfonyl)imide ([VEIM][TFSI]), the interaction between Li⁺ ions and carbonyl groups is weakened by hydrogen bonds and an anti‐coordination effect. This results in a lower Li^+^ transfer barrier and enhanced Li^+^ migration behavior.^[^ ^28^ ^]^

In brief, to facilitate Li^+^ transport kinetics within GPEs, it's necessary to optimize both the ionic conductivity and $t_{Li^{+}}$ simultaneously.

### 3.2 Li^+^ Desolvation Kinetics at Electrode‐Electrolyte Interface and Li⁺ Migration through Electrode/Electrolyte Interphase

The prevailing view is that mass transfer of Li^+^ in the bulk electrolyte constitutes a major obstacle for solid‐state battery operation. Recent research shows that charge transfer at electrolyte‐electrode interfaces and migration through CEI and SEI can be rate‐limiting processes, even for thick electrodes. Boosting Li^+^ desolvation kinetics at electrode‐electrolyte interfaces and Li^+^ migration through the electrode/electrolyte interphase enables better rate performance.

**Figure 12:**
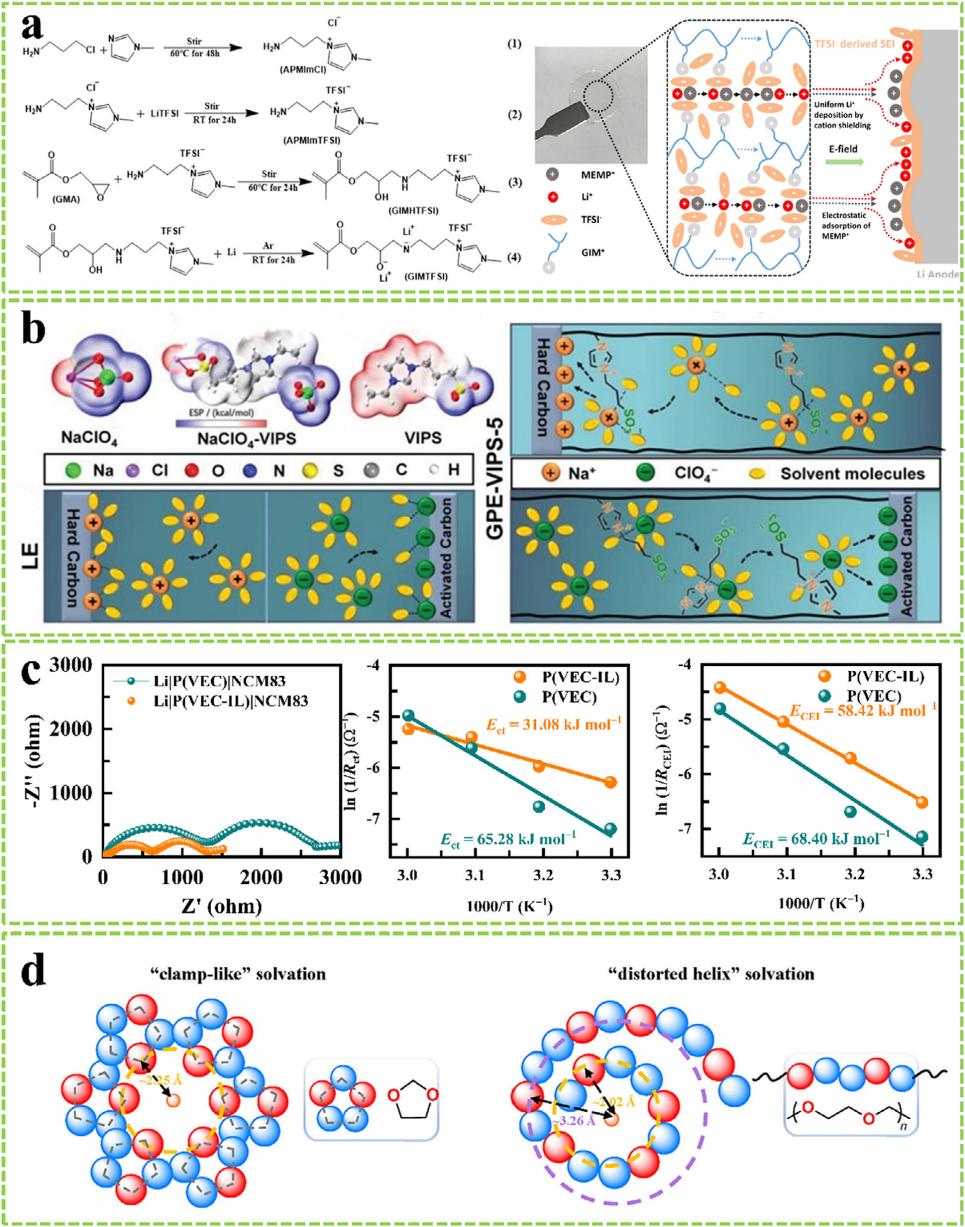
a) Schematic illustration of GPE with enhanced ion conductivity and stable electrode/electrolyte interphase. Reproduced with permission.^[^ ^92^ ^]^ Copyright 2024, American Chemical Society. b) Electrostatic potential distributions of components and schematic illustration of desolvation process in liquid electrolyte and GPE‐VIPS, the curves correspond to the activation energies. Reproduced with permission.^[^ ^93^ ^]^ Copyright 2023, Wiley. c) Activation energies of Li^+^ desolvation process and diffusion through CEI calculated from EIS curves. Reproduced with permission.^[^ ^17^ ^]^ Copyright 2024, Wiley. d) The schematic illustration solvation structures of Li^+^ in 1,3‐dioxolane and Li^+^ in polyacetal.^[^ ^95^ ^]^ Copyright 2023, Elsevier.

Weakening the Li^+^ solvation structure can reduce the interfacial desolvation barrier and contribute to improved rate performance. Introducing Lewis‐acid groups can weaken the Li^+^ solvation structure via electrostatic interactions between functional groups and solvents/anions/polymer matrix. **Figure** **12**a shows that imidazole‐functionalized polymer electrolytes with positive charges and a strong Coulombic effect with TFSI⁻ anions can facilitate solvated Li^+^ desolvation kinetics. N‐methyl‐N‐methoxyethyl‐pyrrolidinium cations can be adsorbed on the anode surface under an electric field, promoting the formation of anion‐derived SEI.^[^ ^92^ ^]^ Zwitterionic GPEs can weaken ion‐solvent interactions and reduce desolvation barriers, enabling fast ion transfer kinetics. Furthermore, the decomposition of 3‐(1‐vinyl‐3‐imidazolio)propanesulfonate (VIPS) contributes to the formation of S‐based and N‐based inorganic interphases on the anode surface, reducing Li⁺ diffusion barriers (Figure 12b).^[^ ^93^ ^]^ Zuo et al. constructed a uniform and highly oxidation‐resistant polymer layer within the inner Helmholtz plane by in situ polymerizing 1‐vinyl‐3‐ethylimidazolium (VEIM) cations preferentially adsorbed on the LiNi_0.83_Co_0.11_Mn_0.06_O_2_ (NCM83) surface, inducing the formation of an anion‐derived cathode/electrolyte interphase with fast interfacial kinetics. The copolymerization of VEIM cations and VEC forms the P(VEC‐IL) copolymer with a positive charge, providing a positive electric field to facilitate Li⁺ transport and desolvation (Figure 12c).^[^ ^17^ ^]^

Tuning the Li^+^ coordination chemistry based on solvent and polymer molecular engineering can improve interfacial kinetics.^[^ ^94^ ^]^ In recent studies, reducing the solvating ability of the solvent or polymer chain can facilitate Li^+^ desolvation. Figure 12d displays a polyacetal electrolyte with alternately changing intervals between ─O─ coordinating sites. Structural asymmetry leads to a unique, distorted helical solvation sheath, which can effectively reduce Li^+^‐electrolyte binding energy and facilitate Li^+^ desolvation kinetics.^[^ ^95^ ^]^

**Figure 13:**
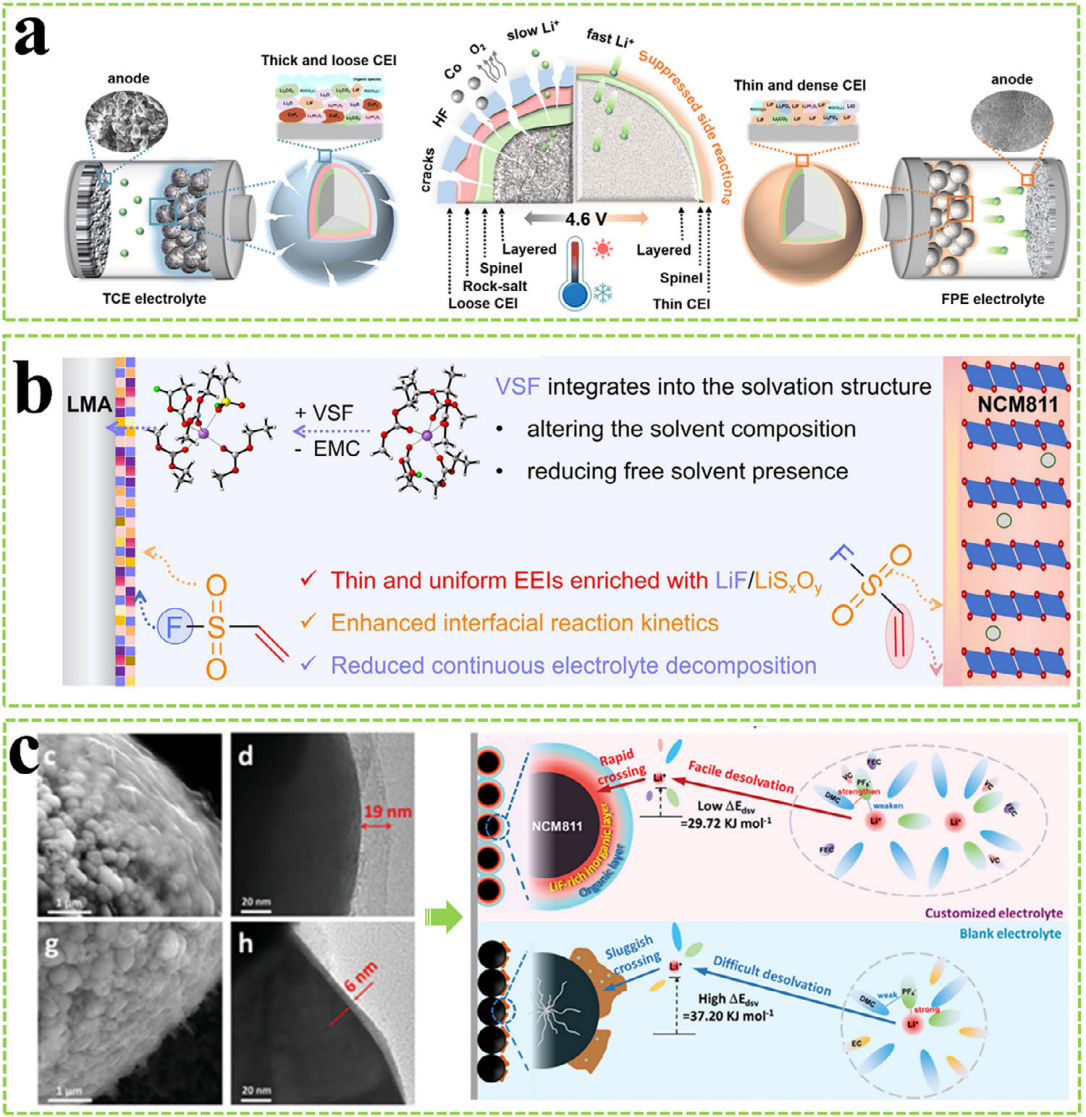
a) Schematic illustration of enhancing Li^+^ transport kinetics. Reproduced with permission.^[^ ^96^ ^]^ Copyright 2024 RSC. b) Schematic of the influence of VSF on the electrode/electrolyte interphase and electrolyte decomposition pathways. Reproduced with permission.^[^ ^97^ ^]^ Copyright 2024, RSC. c) Schematic illustrations of Li^+^ solvation sheath structures in different electrolytes. Reproduced with permission.^[^ ^98^ ^]^ Copyright 2023, Wiley.

Introducing additives or regulating solvation structures can promote the formation of CEI with excellent Li⁺ migration kinetics. Zhang et al. utilized the synergistic cooperation of multi‐component additives to promote the enrichment of inorganic components (LiF and Li_3_PO_4_) with low resistance and high ionic conductivity at the electrode/electrolyte interfaces. This approach suppresses interfacial side reactions and accelerates interfacial reaction kinetics (**Figure** **13**a).^[^ ^96^ ^]^

Ou et al. developed a new bifunctional additive, vinyl sulfonyl fluoride (VSF). The anodic decomposition of the vinyl group forms protective interfacial layers on the cathode, effectively curbing continuous electrolyte degradation and enhancing Li⁺ transport (Figure 13b).^[^ ^97^ ^]^ Xiao et al. formulated a tailored electrolyte to optimize ion desolvation and interphase formation. This electrolyte produces a dual‐layer cathode electrolyte interphase (CEI) comprising an inner inorganic layer rich in LiF and an outer organic layer dominated by ROCOOLi. This structure is both stable and highly efficient for lithium‐ion transport (Figure 13c).^[^ ^98^ ^]^ The aforementioned work highlights that lithium‐ion desolvation and the subsequent cross through the cathode‐electrolyte interphase are also pivotal for high‐rate performance. These critical steps are significantly influenced by the composition of the electrolyte formula.

In this section, we have reviewed the ionic conduction mechanisms and optimization strategies of GPEs to promote Li^+^ diffusion kinetics. We have also discussed approaches to alter the solvation/desolvation activation energies of Li^+^ using solvents, salts, and polymer matrices. In practical applications, extremely fast charging is one of the most significant barriers limiting the widespread adoption of electric vehicles, especially when compared to the rapid refueling of conventional internal combustion engine vehicles. Additionally, batteries still face limitations in electrochemical performance at low temperatures. The primary constraints on the fast charging and low‐temperature electrochemical performance of quasi‐solid‐state lithium batteries can be attributed to charge transfer and mass transport issues. Charge transfer involves the solvation and desolvation of Li^+^ ions and their diffusion across the electrode/electrolyte interfaces, while mass transport refers to the movement of Li^+^ ions within the electrolyte and electrode materials. There is an urgent need to improve both charge transfer and mass transport to alleviate “range anxiety” associated with EVs and enhance electrochemical performance at low temperatures.

## 4 Molecular‐Level Interfacial Chemistry Regulation

To suppress detrimental reactions between the electrolyte and cathode under high voltage, the most common approach is to in situ form an effective CEI (cathode‐electrolyte interphase) on the cathode surface to inhibit the continuous decomposition of the electrolyte. An ideal CEI is expected to be chemically and electrochemically stable. It should be electronically insulative and ionically conductive (only for Li^+^), thus constituting a charge transfer barrier for parasitic reactions between the electrolyte and the positive electrode and enabling the electrolyte to remain stable under high voltage. In this section, three major approaches to suppressing detrimental interactions between the electrolyte and cathode are discussed below.

### 4.1 Solvation Structure Regulation

**Figure 14:**
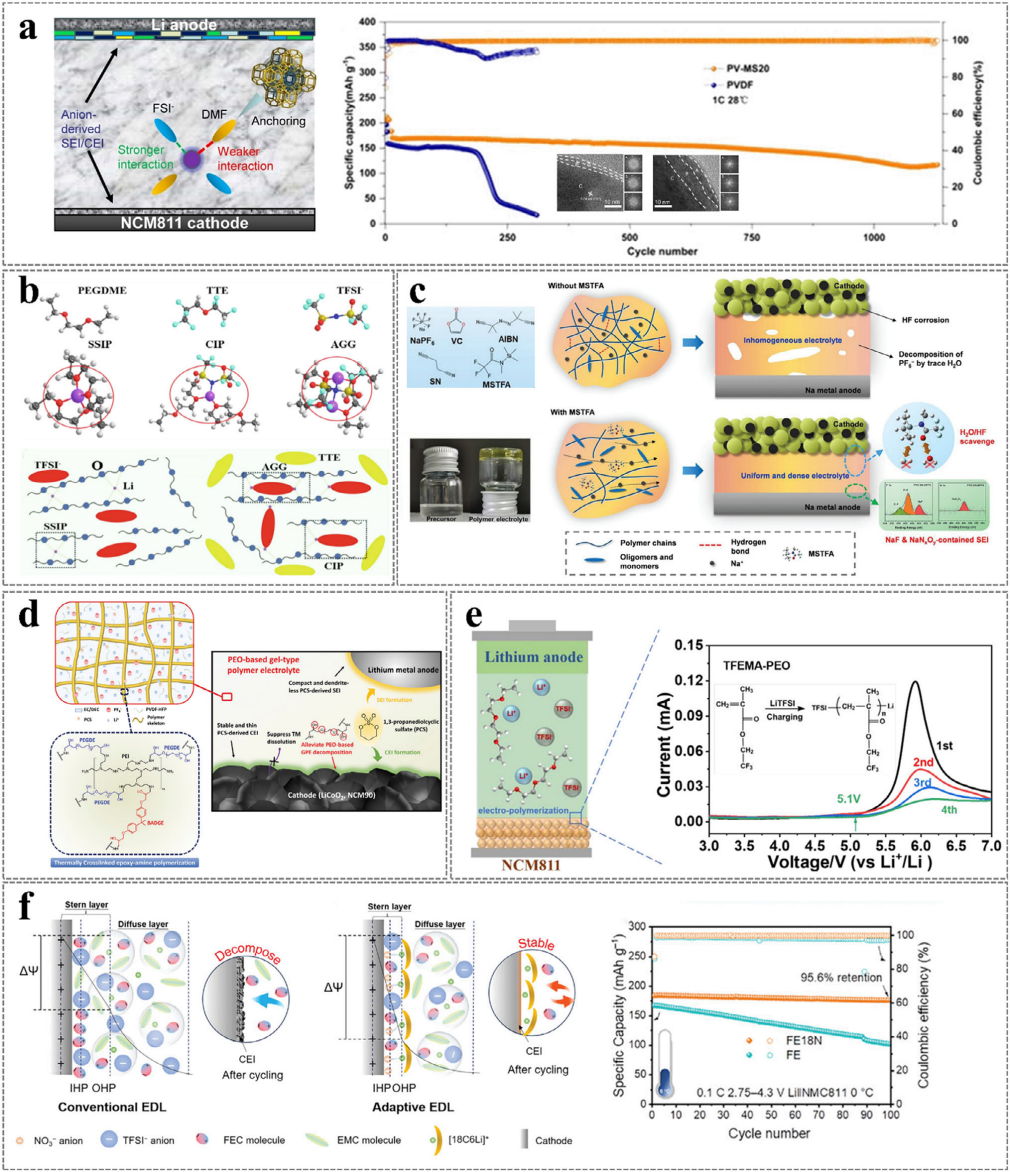
a) Schematic diagram of the working mechanism of solid‐state Li||NCM811 batteries using the PV‐MS20 electrolyte and long‐term cycling performance of Li||NCM811 cells at 2.8−4.3 V at 1 C. The insets are TEM and FFT images of cycled NCM811 cathodes with different electrolytes. Reproduced with permission.^[^ ^101^ ^]^ Copyright 2024, Wiley. b) Li^+^ coordination structures in different electrolytes. Reproduced with permission.^[^ ^102^ ^]^ Copyright 2022, Elsevier. c) Summarization of the multifunction of MSTFA. Reproduced with permission.^[^ ^103^ ^]^ Copyright 2024, Wiley. d) Schematic illustration of the structure and epoxy‐amine crosslinked polymerization of the SA‐TGPE. Reproduced with permission.^[^ ^105^ ^]^ Copyright 2023, Wiley. e) The design principle of ether‐based electrolytes for high‐voltage and high‐safety Li metal batteries. Reproduced with permission.^[^ ^106^ ^]^ Copyright 2022, American Chemical Society. f) Schematic illustration of the structure of EDL behavior constructed at NCM811 surface in FE and FE18N GPE before and after cycling. Reproduced with permission.^[^ ^110^ ^]^ Copyright 2024, Wiley.

The Li^+^ solvation structure can be divided into three categories including solvent‐separated ion pairs (SSIPs), contact ion pairs (CIPs), and aggregates (AGGs) in GPE.^[^ ^99^ ^]^ The anion‐dominated Li⁺ solvation structure is conducive to the formation of a stable and robust CEI, which can promote excellent Li^+^ migration kinetics and inhibit oxidative decomposition of the electrolyte on the surface of high‐voltage cathodes.^[^ ^100^ ^]^ Confining residual solvents or increasing the salt concentration are effective strategies to promote the formation of CIPs and AGGs. Low‐cost 3 Å zeolite molecules can be introduced into GPE to confine residual solvent molecules within the molecular sieve and tailor the solvation structure, leading to more anions coordinated with Li^+^ (**Figure** **14**a).^[^ ^101^ ^]^ A polymeric concentrated GPE (poly‐CGPE) with 10M LiFSI can form a F‐rich confomal cathode eletrolyte interphase on the LiNi_0.5_Co_0.2_Mn_0.3_O_2_ cathode, enabling excellent cycling stability.^[^ ^56^ ^]^ Although the corresponding cycling stability is significantly enhanced in poly‐CGPE, the poor ionic conductivity due to high viscosity severely compromises the electrochemical kinetics. The 1,1,2,2‐tetrafluoroethyl‐2,2,3,3‐tetrafluoropropyl ether diluent was further used to reduce the viscosity of poly‐CGPE while maintaining the merits of the highly concentrated salt, and CIPs and AGGs are generated by leveraging these advantages (Figure 14b).^[^ ^102^ ^]^

### 4.2 Additive Interpahse Engineering

Sacrificial additives undergo preferential oxidation or self‐polymerization on high‐voltage cathode surfaces to establish protective films. Similar to liquid‐state electrolyte additives for high‐voltage cathodes, molecules containing boron, sulfur, nitrogen, and fluorine are often used as film‐forming electrolyte additives for high‐voltage GPEs. To diminish residual gases in GPEs after in situ polymerization, the novel multifunctional additive N‐methyl‐N‐(trimethylsilyl)trifluoroacetamide (MSTFA) was utilized. The HF/H₂O scavenging effect of MSTFA mitigates the corrosion of free acid on the cathode and CEI layer, enhancing cycle stability under high voltage (Figure 14c).^[^ ^103^ ^]^ LiBOB in a trinary salt GPE can form a robust O‐ and B‐rich CEI, inhibiting the continuous degradation of electrolyte solvents and improving oxidative stability.^[^ ^104^ ^]^ 1,3‐propanediolcyclic sulfate incorporated into PEO‐base polymer matrix can form stable interfacial layers on electrodes (Figure 14d).^[^ ^105^ ^]^ Functional residual monomers and polymer matrices can form robust CEI layers through polymerization or synergistic decomposition.^[^ ^36c^ ^]^ Typically, a high‐voltage stable CEI layer based on polyfluoroethyl acrylate was constructed via in situ electropolymerization of unsaturated trifluoroethyl methacrylate (Figure 14e).^[^ ^106^ ^]^ Hierarchical interphases can be constructed by the synergistic decomposition between DFOB^−^ anions and POSS polymer chains, ensuring seamless interfacial contact and enhancing cycling stability.^[^ ^107^ ^]^ The battery is a complex system, and electrolytes affect both the anode and cathode. Complex reactions put forward higher requirements for the functionality of electrolytes, such as improving the stability of the anode.

### 4.3 EDL Engineering Strategies

The electric double layer (EDL), consisting of the inner Helmholtz plane (IHP) and outer Helmholtz plane (OHP), is the region where electrochemical reactions take place between electrodes and electrolytes.^[^ ^108^ ^]^ The specific adsorption behavior in the IHP and the solvation structures in the OHP influence the formation of CEI and SEI layers.^[^ ^109^ ^]^ In zwitterionic GPEs, VIPS can enter the inner Helmholtz layer preferentially and further be reduced to form an inorganic‐rich SEI layer.^[^ ^93^ ^]^ The modified EDL structure, regulated by distinctive [18C₆Li]^+^NO₃^⁻^ cluster adsorption on electrodes, can regulate the interfacial layer composition and construction (Figure 14f).^[^ ^110^ ^]^ Thus, the GPE enables Li||NCM811 cells with excellent cycling stability over a wide operating temperature range. Although substantial evidence has shown that the spatial enrichment/deficiency of components within the EDL plays a dominant role in the formation of the CEI layer, it is imperative to develop advanced characterization techniques to effectively understand the dynamic evolution of the EDL and its impact on the CEI/SEI layer during cycling.

The interaction between the interfacial hydration layer IHL and the passivation film formation process reveals how the concentrated electrolytes can be used to optimize the composition of the CEI. As the concentration of lithium salts increases, the anions become more dominant in the IHLs on both the cathode and anode sides, enhancing the contribution of anion‐derived inorganic components to the electrode passivation films. It is crucial to recognize that the formation of both CEI and SEI depends on the Gibbs free energy difference between the reactants (electrolytes) and the products after electrochemical or chemical reactions. Qualitative trends can be identified by comparing the HOMO and LUMO energies of different electrolyte components.^[^ ^111^ ^]^

In addition to electrolyte composition, the surface chemistry, morphology, and electrode potential of the cathode significantly impact the components and properties of the CEI. The CEI formation is closely tied to the components within the electrical double layers near the electrode before any electrochemical or side reactions. Essentially, the strategies for constructing robust CEIs are interconnected. When certain molecules interact more strongly with electrodes, their adsorption within the IHP and solvation structure in the OHP are altered. Conversely, when molecules prefer solvated coordination, their adsorption on the electrode surface is weakened, which in turn promotes anion adsorption and fosters anion‐induced electrode/electrolyte interfaces, thereby modulating the CEI. Therefore, developing stable CEIs requires a rational integration of multiple strategies.^[^ ^15^ ^]^

Simultaneously, enhancing SEI stability is crucial for the development of high‐voltage lithium batteries.^[^ ^112^ ^]^ Recent reviews have comprehensively summarized various aspects of the SEI, including its formation mechanisms, structure, chemical composition, and conduction mechanisms. Huang et al. have elucidated two critical factors influencing SEI formation: the characteristic adsorption of ions on the electrode surface and the evolution of the solvated coordination structure.^[^ ^15^ ^]^ Sun et al. have provided a detailed review of constructing targeted SEI layers through organic molecular engineering, which offers a promising strategy to tailor SEI properties and thereby improve the electrochemical performance.^[^ ^113^ ^]^

## 5 Safety Considerations in High‐Energy‐Density Lithium Batteries

Higher energy‐density lithium batteries with high‐voltage cathodes inevitably exacerbate safety concerns. Currently, a considerable amount of work has been dedicated to preventing thermal runaway in lithium batteries. The monomer conversions of GPEs through in situ polymerization range from 10% to 90%, resulting in 10% to 90% of liquids remaining.^[^ ^114^ ^]^ Under elevated temperatures, a dense crosslinked polymer network can be spontaneously formed through the polymerization of residual monomers incorporating abundant active unsaturated double bonds or cyclic ethers. Additionally, nucleophilic or electrophilic additions involving chemistries, including solvents, salts, and functional groups in the polymer skeleton, can further enhance this network.^[^ ^115^ ^]^ This dense network can slow down the diffusion kinetics of active gases and lithium ions. Designing “safe” solvents, lithium salts, and smart risk‐responding polymer backbones remains crucial for developing high‐safety GPEs.^[^ ^116^ ^]^

## 6 Summary and Outlook

**Figure 15:**
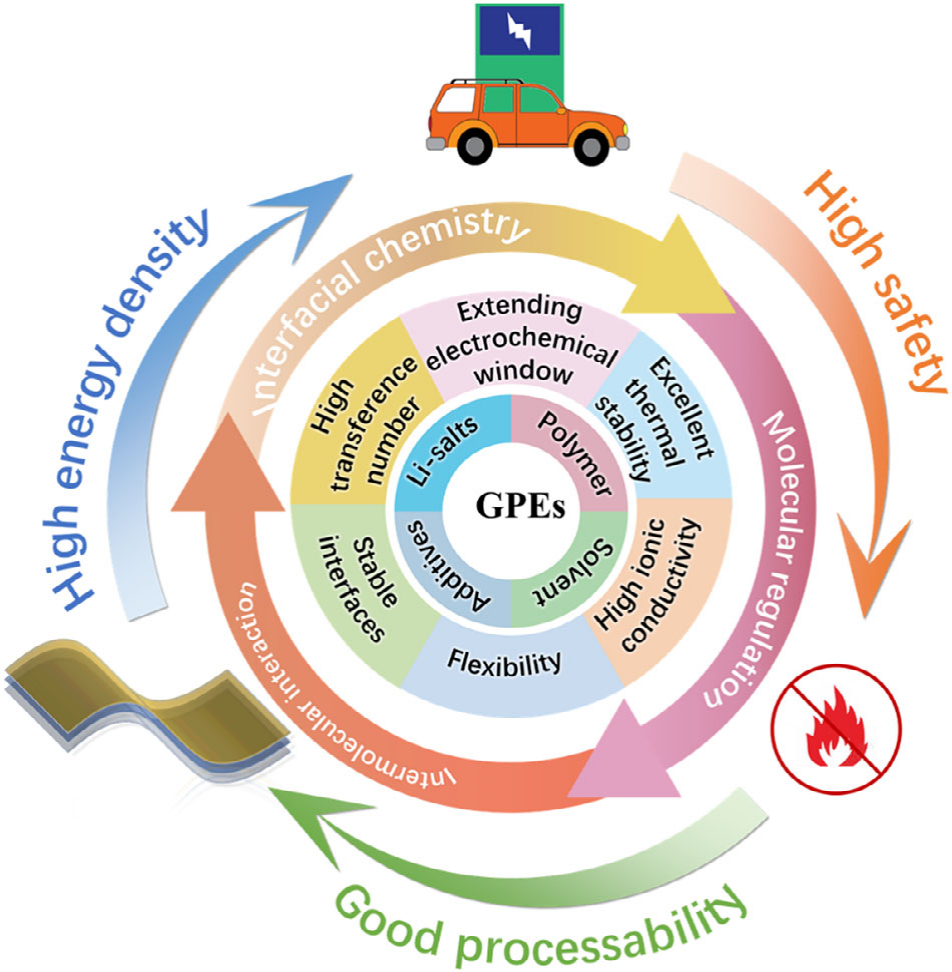
Schematic illustration of molecular regulation and intermolecular interactions in GPE.

In this review, we present recent progress in improving high‐voltage stability and Li⁺ transport kinetics in gel polymer electrolytes through molecular regulation and intermolecular interactions. The delocalization of electron cloud density, caused by electron‐withdrawing substituents or intermolecular interactions, results in enhanced oxidative stability. The Li^+^ transport kinetics, including the transport of solvated Li^+^ ions in GPEs, the desolvation process of Li^+^ ions at electrode/electrolyte interfaces, and Li^+^ ion migration through electrode/electrolyte interphases, can be improved by regulating the solvating capability of GPEs (**Figure** **15**).

The development of high‐voltage GPEs may involve the combinatorial optimization of polymer matrices, lithium salts, and solvents. Polymer hosts containing electron‐withdrawing functional groups can enhance high‐voltage stability. Meanwhile, incorporating plasticizers such as ionic liquids, fluorinated solvents, or nitrile solvents can boost the ionic conductivity of the GPE without sacrificing high‐voltage performance. Moreover, lithium salts with higher dissociation energies or those that favor the formation of a robust CEI can further improve ionic conductivity and cycling stability at elevated voltages. Regarding the future development of high‐voltage GPE for high‐energy‐density lithium batteries, the following proposed perspectives should be prioritized:

1. Artificial intelligence‐assisted multiscale design of GPE. Traditional approaches, which rely heavily on trial‐and‐error experimentation, are time‐consuming and resource‐intensive. Artificial intelligence (AI)‐driven algorithms can analyze vast datasets by identifying complex patterns and relationships, thereby predicting the optimal composition and structure of electrolytes and significantly accelerating the discovery process. For instance, machine learning models can simulate the interactions between different electrolyte components and electrodes, optimizing properties such as ionic conductivity and electrochemical stability. This not only enhances the efficiency of electrolyte development but also opens new avenues for creating bespoke materials tailored to specific battery applications. As AI continues to evolve, its integration into electrolyte design promises to unlock unprecedented advancements in battery technology, driving the transition to a more sustainable and efficient energy future. Several challenges should be tackled, for instance, defining widely accepted standards in GPE combined with systematic data disclosure, identifying the most suitable descriptor(s) for a certain machine learning model, or determining the associated error, among others.
2. Investigating the self‐healing characteristics. Continuous electrochemical cycling and mechanical deformations cause inferior electrode/electrolyte interfacial contact. In situ polymerized self‐healing GPE with excellent adhesive capability is quite significant for forming conformal interfaces that synchronously elongate and shrink with the electrodes. Therefore, maintaining molecular‐level intimate and compatible electrode/electrolyte interfaces and interparticle interfaces in a continuously dynamic state is crucial. In addition to self‐healing characteristics, an ideal self‐healing polymer electrolyte should possess fast Li⁺ transport kinetics, a faster healing time, good mechanical properties, low interfacial resistance between the electrolyte and electrodes, and a wide electrochemical stability window.
3. Developing advanced characterization methods. Understanding the complex interactions at the electrode/electrolyte interface, especially under high‐voltage conditions, is crucial for developing high‐performance batteries. Tracking the interfacial evolution and decoupling the multiple reactions that occur at high voltage are essential steps in this process. Advanced characterization techniques play a pivotal role in providing detailed insights into these dynamic processes. In situ observations and multiple characterizations, such as in situ mass spectrometry, in situ Raman spectroscopy, in situ Fourier‐Transform Infrared spectroscopy (FT‐IR), nuclear magnetic resonance (NMR) spectroscopy, and computed tomography, offer powerful tools for monitoring the formation and dynamic evolution of electrode/electrolyte interphases. These techniques enable real‐time tracking of ion transport across interfaces, providing valuable data on the mechanisms of interfacial reactions and the stability of the interphases under various operating conditions.
4. Screening monomer and optimizing common initiation conditions. In situ polymerized solid‐state batteries are a highly promising manufacturing method for high‐energy lithium batteries, without separating and purifying the polymer. Cationic polymerization methods, primarily utilizing cyclic ether monomers, provide excellent monomer conversion rates without inducing impurities. However, polyethers exhibit narrow electrochemical windows and are susceptible to oxidation at high voltages. This limits their ability to form stable cathode solid electrolyte films and effectively match high‐potential cathode materials. Therefore, exploring new monomers with enhanced high‐voltage stability is imperative. For free radical polymerization, the choice and concentration of initiators, such as AIBN, must be carefully controlled due to potential reactivity with Li metal electrodes. A suitable polymerization temperature is also essential to prevent damage to electrode materials and ensure optimal performance of solid‐state polymer lithium batteries. Additionally, the presence of residual monomers in in situ polymerized GPE is a significant issue in real‐world applications, as monomers with reactive functional groups may continue to decompose during battery operation.

## Conflict of Interest

The authors declare no conflict of interest.
